# Proteases as Therapeutic Targets Against the Parasitic Cnidarian *Ceratonova shasta*: Characterization of Molecules Key to Parasite Virulence In Salmonid Hosts

**DOI:** 10.3389/fcimb.2021.804864

**Published:** 2022-01-07

**Authors:** Gema Alama-Bermejo, Pavla Bartošová-Sojková, Stephen D. Atkinson, Astrid S. Holzer, Jerri L. Bartholomew

**Affiliations:** ^1^ Institute of Parasitology, Biology Centre, Czech Academy of Sciences, České Budějovice, Czechia; ^2^ Department of Microbiology, Oregon State University, Corvallis, OR, United States

**Keywords:** myxozoa, cysteine protease, aspartic protease, aminopeptidase, stefin, 3D protein structure, gene expression, homologous search

## Abstract

Proteases and their inhibitors play critical roles in host-parasite interactions and in the outcomes of infections. *Ceratonova shasta* is a myxozoan pathogen that causes enteronecrosis in economically important salmonids from the Pacific Northwest of North America. This cnidarian parasite has host-specific genotypes with varying virulence, making it a powerful system to decipher virulence mechanisms in myxozoans. Using *C. shasta* genome and transcriptome, we identified four proteases of different catalytic types: cathepsin D (aspartic), cathepsin L and Z-like (cysteine) and aminopeptidase-N (metallo); and a stefin (cysteine protease inhibitor), which implied involvement in virulence and hence represent target molecules for the development of therapeutic strategies. We characterized, annotated and modelled their 3D protein structure using bioinformatics and computational tools. We quantified their expression in *C. shasta* genotype 0 (low virulence, no mortality) and IIR (high virulence and mortality) in rainbow trout *Oncorhynchus mykiss*, to demonstrate that there are major differences between the genotypes during infection and parasite development. High proliferation of genotype IIR was associated with high expression of the cathepsin D and the stefin, likely correlated with high nutrient demands and to regulate cell metabolism, with upregulation preceding massive proliferation and systemic dispersion. In contrast, upregulation of the cathepsin L and Z-like cysteine proteases may have roles in host immune evasion in genotype 0 infections, which are associated with low proliferation, low inflammation and non-destructive development. In contrast to the other proteases, *C. shasta* aminopeptidase-N appears to have a prominent role in nematocyst formation in both genotypes, but only during sporogenesis. Homology searches of *C. shasta* proteases against other myxozoan transcriptomes revealed a high abundance of cathepsin L and aminopeptidase homologs suggesting common gene requirements across species. Our study identified molecules of potential therapeutic significance for aquaculture and serves as a baseline for future research aimed at functional characterisation of these targets.

## Introduction

Host-parasite interactions involve an arsenal of different molecules, including proteases and their inhibitors, which are ubiquitous in parasites. These molecules have central roles in parasite life cycles, including invasion, migration, feeding and immunomodulation of host responses (McKerrow et al., 2006; Klotz et al., 2011), and represent attractive targets for drug design as antiparasite intervention strategies (e.g. Coombs et al., 2001). Proteases and their inhibitors are considered parasite virulence factors and regulators of disease pathogenesis (e.g. *Entamoeba* spp., *Trypanosoma* spp. and other protozoans, Serrano-Luna et al., 2013; Siqueira-Neto et al., 2018). Destruction of host extra-cellular matrix and other proteins including immune factors, are some of the pathogenic functions that proteases can have during parasitic infections (e.g. *Fasciola hepatica*, Dalton et al., 2003; *Leishmania mexicana*, Cameron et al., 2004 and other protozoans, Piña-Vázquez et al., 2012). Differences in protease expressions exist between parasite genotypes or strains with defined virulence phenotypes (Davis et al., 2007) and these differences can help to identify specific proteases representing candidate virulence genes.

Myxozoans are microscopic cnidarian endoparasites that underwent morphological simplification from their free-living cnidarian ancestors almost 600 million years ago (Holzer et al., 2018). These parasites alternate between an invertebrate host, annelids or bryozoans, and a vertebrate host, usually fish; in each host they produce spores as waterborne transmission stages. Myxozoans cause important economic losses in aquaculture and wild fishes, and are an increasing concern as causative agents of emerging diseases, under expanding aquaculture and global climate change (Videira et al., 2016; Strepparava et al., 2018). For example, predictions indicate that climate change will transform endemic or low risk infections for the myxozoan *Ceratonova shasta* to high disease risk in Chinook salmon at the Klamath River (USA) (Ray et al., 2015). No effective treatments are available against myxozoan infections, and design of efficient therapeutic strategies is limited by a lack of knowledge regarding host-myxozoan interactions. Initial findings include unique cysteine and serine proteases identified from lysates of spore-forming plasmodia of myxozoan species that degrade their host tissues enzymatically (e.g. Martone et al., 1999; Kelley et al., 2004; Funk et al., 2008), suggesting these molecules as potential targets for therapeutic intervention. Recent availability of genomic and transcriptomic datasets of myxozoans (reviewed in Alama-Bermejo and Holzer, 2021) have allowed identification of large families of proteases, e.g. 2.5% of proteins (422 proteases) in the *Thelohanellus kitauei* genome (Yang et al., 2014), 2.6% (235 proteases) in the *Sphaerospora molnari* trancriptome (Hartigan et al., 2020) or 6.1% (7 proteases) in the *Ceratonova shasta* nematocyst proteome (Piriatinskiy et al., 2017). It further has been demonstrated that these proteins are genetically diversified (Eszterbauer et al., 2020) or structurally modified (Hartigan et al., 2020; Bartošová-Sojková et al., 2021) in some myxozoan species, underlining the importance and specificity of these molecules in parasite survival and successful host interaction. Thus, the quantity and diversity of proteases in myxozoans opens a world of possibilities for exploring them as potential drug targets.

*Ceratonova shasta* (syn. *Ceratomyxa shasta*) is a myxozoan parasite that can cause acute enteronecrosis in economically and ecologically valuable salmon species in the rivers of the Pacific Northwest of North America. *C. shasta* infection initiates when actinospores invade the fish through the gills, and the parasite migrates to the target organ, the intestine (Bjork and Bartholomew, 2010). Once in the intestine, it proliferates and invades all tissue layers, promoted by high adhesiveness and motility of proliferative stages (Alama-Bermejo et al., 2019). *C. shasta* is a unique myxozoan because it occurs as host-associated genotypes (0, I, IIC, IIR) that differ in virulence (Atkinson and Bartholomew, 2010; Stinson et al., 2018; Breyta et al., 2020; Alama-Bermejo et al., 2020). Infections in rainbow trout by genotypes 0 and IIR are contrasting systems that can be used to decipher mechanisms of virulence in myxozoans (Alama-Bermejo et al., 2019; Alama-Bermejo et al., 2020; Taggart-Murphy et al., 2021). Genotype 0 causes a chronic low-virulence infection, characterized by minimal proliferation and delayed spore production, with no mortality (Alama-Bermejo et al., 2019). In contrast, genotype IIR causes a highly virulent infection, characterized by rapid and massive proliferation with up to 100% mortality within 1 month after exposure. Using this model, we pinpointed key differences in expression of parasite motility and adhesion genes, which suggested different exploitation, migration and proliferation between genotypes (Alama-Bermejo et al., 2019).

In a previous study, we identified the importance of proteases in *C. shasta* virulence by demonstrating high pressure for genetic modification *via* mutations and SNP polymorphism in relation to virulence (Alama-Bermejo et al., 2020). In this study, we focused on four proteases belonging to three different catalytic types: cysteine, aspartic, and metallo- proteases, and one cysteine protease inhibitor. We quantified and compared their differential expression over time in low (0) and high (IIR) virulence *C. shasta* genotype infections in rainbow trout by specific qPCR assays. Using genomic and transcriptomic evidence, we modeled secondary and tertiary structures by homology detection and prediction approaches and performed a specific homology search of proteases in available myxozoan species transcriptomes. From the data obtained we discuss potential roles of these molecules during *C. shasta* infection, and compare them to known proteases in myxozoans and other parasite groups. Knowledge of parasite proteases, their gene expressions and protein structures is essential to better understand their roles in host-parasite interaction and the mechanisms underlying resistance, tolerance and virulence.

## Materials and Methods

### Gene Mining in *C. shasta* IIR Genome and Transcriptome

Proteases and inhibitors were mined from our host-free *C. shasta* IIR reference transcriptome (Alama-Bermejo et al., 2020; NCBI SRA Acc. number SRR6782113 and Dryad database doi:10.5061/dryad.tx95x69tt). Gene annotations were confirmed by BLAST (blastx, accessed October 2019) searches against UniProt (accessed August 2019), Gene Ontology (GO, last accessed August 2019), Conserved Domains Database CDD (NCBI, last accessed October 2019) and MEROPS, the peptidase database (http://merops.sanger.ac.uk/; Rawlings et al., 2018, last accessed October 2019). To obtain KEGG orthology (KO) annotation, BlastKOALA was used (https://www.kegg.jp/blastkoala/; Kanehisa et al., 2016; last accessed May 2020). Based on literature, the following protease/inhibitor genes (**Table 1**) were selected because of their known roles in the life cycle of other parasites and/or pathogens: 1) aspartic protease: cathepsin D (Sojka et al., 2016), 2) cysteine proteases: cathepsin-L and cathepsin Z-like (or cathepsin X) (Kelley et al., 2003; Grote et al., 2018), 3) zinc-metalloprotease: aminopeptidase-N (Gras et al., 2014) and 4) cysteine protease inhibitor: type 1 cystatin (stefin, Ranasinghe and McManus, 2017, Bartošová-Sojková et al., 2021). Using the transcriptomic sequence of these genes, we searched for them in genomic sequence in *C. shasta* IIR genome (100bp paired reads Illumina HiSeq 2000, Assembly version Velvet 2015-93, 14,586 sequences, 185-452,519 bp length, N50 = 36,283, total size 69.8 Mb; European Nucleotide Archive Project Accession Number PRJEB48361) to determine the organization of these genes at both DNA and RNA levels. To confirm the genes were of parasite origin, we designed gene-specific primers using NCBI/Primer-BLAST (Ye et al., 2012; **Table 1**) and conducted PCR and amplicon sequencing on DNA and cDNA of type IIR and 0 infected and non-infected rainbow trout samples. PCR protocols, purification and sequencing was performed as described in Atkinson et al. (2018) and Alama-Bermejo et al. (2019).

### *C. shasta* Genotype 0 and IIR Infections for Gene Expression Analyses

Experimental infections of rainbow trout *Oncorhynchus mykiss* by *C. shasta* genotypes 0 and IIR were carried out as described in Alama-Bermejo et al. (2019). Briefly, SPF rainbow trout from Roaring River Hatchery (Scio, OR, Oregon Department of Fish and Wildlife, USA) (length 5.5–7.5 cm; weight 1.6–3.8 g, age 0 approx. 6 months old) were exposed for 72 h in two locations where each genotype occurs in the Klamath River, OR/CA, USA. To characterize parasite exposure, water samples (3x 1L) collected from each location were filtered and quantified using an absolute SSU rDNA-based qPCR assay; genotype was confirmed by ITS-1 rDNA region sequencing. These assays are routinely used in Bartholomew’s lab and are well documented in previous publications (Hallett and Bartholomew, 2006; Atkinson and Bartholomew, 2010; Hallett et al., 2012; Atkinson et al., 2018). Both groups of fish were kept at 18°C in well water at the Aquatic Animal Health Laboratory at Oregon State University (AAHL, OSU). At days 7, 15, 22 and 29 post-exposure (dpe), 5 fish per group were euthanized using the anesthetic MS222 (Argent Chemical Laboratories, Redmond, WA, USA). Intestines were sampled and portions of each were stored frozen at -20°C for DNA, in RNAlater for RNA (Ambion, Austin, TX, USA), and examined in wet mount using light microcopy; parasite infection was recorded and fish mortalities were monitored. SPF rainbow trout (n=5) from the same stock, kept under the same conditions at the AAHL, were sampled as negative infection controls. Intestine DNA samples were extracted using DNeasy Blood and Tissue kit (Qiagen, Valencia, California). Parasite genotype was confirmed by PCR assay (Atkinson and Bartholomew, 2010; Atkinson et al., 2018). Parasite quantification was performed using a modified *C. shasta* SSU rDNA qPCR assay (Hallett et al., 2012; Alama-Bermejo et al., 2019). All sequencing and genotyping in this study was performed using ABI BigDye Terminator Cycle Sequencing Kit v3.1 and ABI3730 Genetic Analyzer (Applied Biosystems, Foster City, California, USA) at the Center for Quantitative Life Sciences, CQLS, OSU. Each genotype had markedly different infection dynamics, parasite proliferation, clinical signs and mortality (see Alama-Bermejo et al., 2019 for further details).

### Gene Expression Assays on Genotype 0 and IIR Infections

Intestinal RNA was extracted using the High Pure RNA tissue kit (Roche, Basel, Switzerland), including a DNase step, and quantified using NanoDrop at the CQLS. Detection of genomic DNA contamination and quality of RNA was determined as described in Alama-Bermejo et al. (2019). cDNA was synthesized using Transcriptor High Fidelity cDNA Synthesis Kit (Roche) using 500 ng of starting RNA and anchored-oligo (dT)18 primer.

For relative quantitation, we used three *C. shasta* reference genes GAPDH, NADH and HPRT-1 (Alama-Bermejo et al., 2019). Primer amplification efficiencies of target and reference genes were obtained using a set of a 2-fold serial dilutions between 0.625 ng/μL and 5 ng/μL and run using the qPCR assay described below. Efficiencies were obtained using the slope of the standard curve in the StepOne software (Applied Biosystems), allowing ±10% variation (**Table 1**).

qPCR and relative gene expression analyses were performed as described in Alama-Bermejo et al. (2019). Briefly, five fish intestines per genotype and sampling time point (n=50) and three intestines from uninfected control rainbow trout in total were analyzed for the selected proteases and inhibitor genes. The qPCR reaction mix comprised TaqMan Universal PCR Master Mix (Applied Biosystems) and SYTO9 Green Fluorescent Nucleic Acid Stain (Molecular Probes, USA) as fluorophore, with 5 ng of cDNA. All reactions were simplex and run in triplicate in a StepOnePlus Real Time PCR system (Applied Biosystems). Cycling conditions consisted of a polymerase activation at 50 °C for 2 min, denaturation at 95 °C for 10 min, 44 cycles of 95 °C for 15 s and annealing at 60 °C for 1 min, and melting curve analysis for detection of any nonspecific PCR products (95 °C for 15 s, 64 °C for 60 s and a step interval of +0.3 °C every 15 s until 88 °C was reached). All plates were run with an inter-plate calibrator (a positive *C. shasta* sample with NADH assay), a no-template control, and ROX as the passive reference. Cq mean was calculated for each sample and inter-plate correction applied when necessary (Cq +/- 0.5). Relative gene expression was calculated as fold change using 2^-ΔΔCq^ method (Schmittgen and Livak, 2008), being ΔΔCq=[(Cq gene of interest – Cq average three reference genes) genotype IIR or ¨treated¨ group – (Cq gene of interest – Cq average three reference genes) genotype 0 or ¨calibrator¨ group]). Additionally, intragenotype temporal changes were calculated as relative change (2^-ΔCq^) to the reference genes. Differences in fold and relative changes were analyzed for statistical significance with SigmaPlot 13.0 (Systat Software, Inc, USA) using Tukey´s method for multiple comparisons after one-way ANOVA or t-test for normally distributed data. For non-normally distributed data, a Kruskal-Wallis with Dunn’s multiple comparison was performed.

### Analyses of Amino Acid Sequences and Protein Structure Predictions

The secondary and 3D structures of the selected *C. shasta* proteins were predicted to obtain more information about structural differences to known models and their potential effect on function. Using ORFinder (https://www.ncbi.nlm.nih.gov/orffinder/; Rombel et al., 2002) the RNA sequences of the five candidate genes were translated into protein sequences. Presence of any signal peptide was predicted using SignalP 4.1 (Nielsen, 2017) and if present, was removed before running protein structure predictions. Protein structures were predicted by thread modelling approach from the automatically chosen structure templates in the intensive mode of PHYRE2 Protein Fold Recognition Server (Kelley et al., 2015). The templates for the final 3D structure modeling were selected based on heuristics to maximise confidence, percentage identity and alignment coverage. In this program, the superposition of the secondary structure alignments of *C. shasta* proteins with their closest template sequences was also performed. Superposition of the newly generated *C. shasta* models with the closest crystal structures was performed using the UCSF Chimera server (Pettersen et al., 2004). The stereo-chemical qualities of the protein structures were evaluated by Ramachandran plots in PROCHECK 3.4 (Laskowski et al., 1993) implemented within the SAVES webserver (Structural Analysis and Verification Server; https://saves.mbi.ucla.edu/).

### Protease Gene Mining in Myxozoans Transcriptomes

The four protease protein sequences were used as queries to find homologous genes in seven myxozoan transcriptomes: *Tetracapsuloides bryosalmonae*, *Sphaerospora molnari*, *Myxobolus cerebralis*, *Myxobolus squamalis*, *Henneguya salminicola* and *Kudoa iwatai* to compare their protease repertoire with that of our model organism transcriptome, *C. shasta* (genotype IIR). Similar searches were done for myxozoan stefins in a previous publication (Bartošová-Sojková et al., 2021) and are discussed herein. Transcriptomes were downloaded from public data repositories (figshare, Dryad and Transcriptome Shotgun Assembly Sequence Database or TSA at NCBI, see **Table 2**). Protein coding regions within transcript sequences were predicted using default settings in program ‘LongestOrfs’ and ‘Predict’ implemented in TransDecoder v5.5.0. Homologous sequences were searched using BlastP (NCBI) and results were parsed using bitscore >100. Candidate sequences were then searched against NCBI Non-Redundant (NR) database using BlastN to identify any remaining host contamination and to the MEROPS database, which consists of a non redundant library of full-length sequences of peptidases and peptidase inhibitors (https://www.ebi.ac.uk/merops/, Release 12.1, Downloaded July 2020; Rawlings et al., 2018) for confirmation of their identity as proteases. Myxozoan candidate sequences were then aligned to *C. shasta* proteases using Geneious alignment with default settings in Geneious Prime 2020.0.3 (Biomatters, Auckland, New Zealand) to confirm their homology and shared conserved domains (see searches results and sequences in **Supplementary File 2**).

## Results

### Virulent Genotype Upregulates Stefin, Cathepsin D, and Aminopeptidase-N, but Downregulates Both Cysteine Proteases

**Figure 1:**
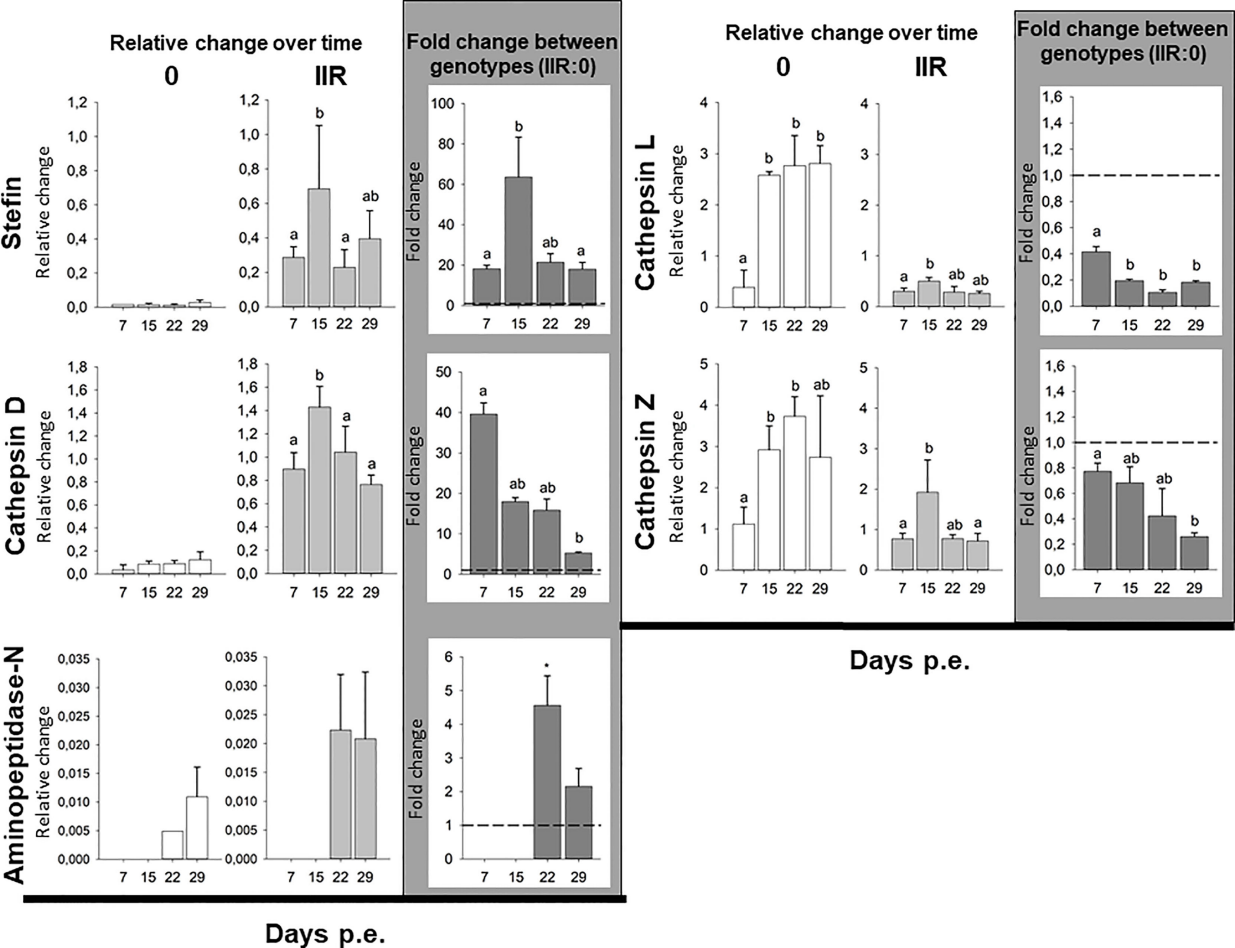
*Ceratonova shasta* proteases and stefin gene expression in the intestine: relative change (2^-ΔCq^) for each genotype over time and fold change (2^-ΔΔCq^) between genotypes (IIR:0) using three reference genes (GAPDH, NADH and HPRT-1).

Gene expression showed highly different trends between genotypes. The stefin had extremely low levels of expression in the low virulent genotype 0 throughout the whole course of infection, while it was variably expressed in the virulent type IIR, with the peak of expression at 15 dpe, and to a lesser extent at 29 dpe. Similarly, cathepsin D expression was comparably low in all time points in type 0. In contrast, high expression of this protease was observed across the whole course of infection in IIR, with a significant peak of expression at 15 dpe, and a slight decrease later on the infection. Aminopeptidase-N had a very distinctive pattern of expression over time in both genotypes with expression detected only during later stages of infection (22 & 29 dpe). Expression of this gene was only fully quantified in replicate fish at 29 dpe for type 0 (only one type 0 fish at 22 dpe), and showed similar expression for IIR at both time points (22 & 29 dpe). Unlike stefin, cathepsin D and aminopeptidase-N, both cysteine proteases were highly expressed by genotype 0 throughout the infection, with the highest relative change observed in this study. Cathepsin L in type 0 had a significant increase at 15 dpe and onwards in the infection. Cathepsin-Z showed a similar trend in type 0, with high expression that increased over time. Cysteine proteases showed low expression levels for genotype IIR throughout the infection, with a small increase at 15 dpe (**Figure 1** and **Supplementary Table 1**).

Comparison of proteases and inhibitor gene expression between each genotype infecting the intestines of rainbow trout revealed that three genes, i.e. stefin, cathepsin D and aminopeptidase-N were upregulated in the virulent rainbow trout IIR. The highest fold changes between genotypes were observed for stefin (up to 63-fold change) and cathepsin D (up to 39-fold change). Stefin and cathepsin D were upregulated at all time points in IIR infections. Stefin had an 18 to 20-fold upregulation in IIR, with a peak at 15 dpe (63-fold change). Cathepsin D had an early peak at 7 dpe with a 39-fold change. Aminopeptidase-N was only upregulated late in the infection, at 22 and 29 dpe, with 4- and 2-fold changes, respectively. Both cysteine proteases were downregulated in genotype IIR throughout the infection (**Figure 1** and **Supplementary Table 2**).

### *In Silico* Characterization, Annotation, and Structural Modeling of *Ceratonova shasta* IIR Proteases and Stefin

#### Cathepsin D

**Figure 2:**
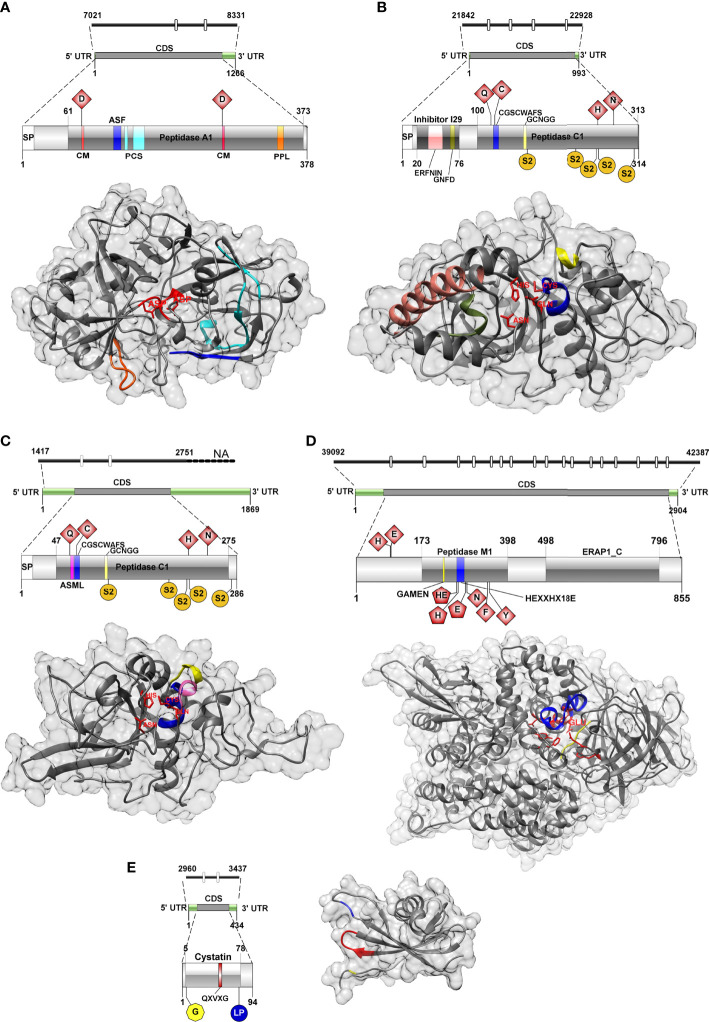
Genomic, transcriptomic and predicted 3D structure of IIR *Ceratonova shasta* proteases and stefin analyzed in this study. **(A)** Aspartic protease, **(B)** Cathepsin L, **(C)** Cathepsin Z, **(D)** Aminopeptidase-N, **(E)** Stefin. Cathepsin D, catalytic DTG/DSG motifs with catalytic sites (CM), red; active site flap (Y flap, ASF), blue; posttranslational cleavage site (PCS), cyan; polyproline loop, orange; Cathepsin L, catalytic sites, red; ERFNIN motif, light pink; active site mini loop (ASML), dark pink; GNFD motif, dark green; CGSCWAFS motif, blue; GCNGG motif, light yellow; S2 subsites, dark yellow; Cathepsin Z, catalytic sites, red; active site mini loop, dark pink; CGSCWAFS motif, blue; GCNGG motif, light yellow; S2 subsites, dark yellow; Aminopeptidase-N, active sites, red (pentagon shaped active sites (in 2D) were also annotated as Zinc binding sites); GAMEN motif, yellow; HEXXHX_18_E motif, blue; Stefin, N-terminal glycine, yellow; QXVXG motif, red; LP pair, blue.

The genome region encoding *C. shasta* IIR cathepsin D was 1,310 bp and contained two introns (22 and 23 bp). Two gene isoforms were detected in the transcriptome showing 3 bp differences (**Supplementary Tables 3** and **4**), and 1,137/1,266 bp corresponded to the coding sequence (CDS) from which a 378 aa ORF was predicted. The gene isoforms showed 3 aa changes at positions 38, 40 and 43, in a region with no predicted conserved catalytic domains. Functional annotations of *C. shasta* cathepsin D suggested it represents a protease of the family A1 or the pepsin family, with an aspartic-type endopeptidase activity, which is involved in proteolysis in the lysosome pathway (**Table 1**). Basic primary organization of the predicted protein (**Figure 2A** and **Supplementary Table 5**) involves a signal peptide, a peptidase A1 domain with two catalytic residues (Asp^79^, Asp^265^) in the typical aspartic conserved motifs (DTG, DSG), an active site flap (Y flap loop) and a posttranslational cleavage site.

The 3D model of *C. shasta* cathepsin D (**Figure 2A**) was constructed based on six templates sharing 36-40% sequence identity to the template residues. 99% of residues were modelled at 100% confidence and only four residues, positioned at the N- and C-terminus, were modelled *ab initio* (**Supplementary Table 6**). The stereo-chemical quality analysis of the 3D structure of *C. shasta* cathepsin D possessed 87% of residues in the most favored regions (see Ramachandran plot in **Supplementary Figure 1A**). The comparison of the secondary structure alignment and the superposition of the 3D structures of *C. shasta* and tick cathepsin D (PDB: 5N7N) zymogens showed a strong overlap in their structures (**Supplementary Figure 1B**). As for the conservative regions important for substrate binding, a structural match was found in the Y flap loop and the active site cleft motifs (DTG, DSG) while the polyproline loop of *C. shasta* cathepsin D had a longer coil region due to the presence of three additional serines (**Supplementary Figure 1C**).

#### Cathepsin L

The genomic sequence encoding cathepsin L was 1,086 bp and contained 4 introns (23-24bp). Only a single isoform was recovered from the transcriptome (**Supplementary Tables 3** and **4**). CDS was 945 bp over a 993 bp transcript and corresponded to a 314 aa ORF. Functional annotation indicated a protease belonging to the family C1 (papain family), with cysteine-type peptidase activity, potentially involved in proteolysis in the lysosome, phagosome and/or during antigen processing and presentation pathways (**Table 1**). The primary organization of *C. shasta* cathepsin L (**Figure 2B** and **Supplementary Table 5**) revealed a signal peptide, an I29 pro-peptide inhibitor domain and a C1 peptidase domain, with 4 active sites (Gln^118^, Cys^124^, His^260^, Asn^281^) and five S2 subsites (Phe^166^-Pro^167^, Val^232^, Met^258^, Ala^261^ and Met^308^), which compose the S2 pocket.

The 3D model of *C. shasta* cathepsin L (**Figure 2B**) was constructed based on six templates sharing 35-39% identity with the target molecule. All residues were modelled at 100% confidence (**Supplementary Table 6**). The stereo-chemical quality analysis predicted that *C. shasta* cathepsin L model had 86% residues positioned in the most favored regions (**Supplementary Figure 2A**). The comparison of the secondary structure alignment and the superposition of the 3D structures of *C. shasta* cathepsin L zymogen and *Fasciola hepatica* procathepsin L1 (PDB: 2O6X) showed a strong structural overlap (**Supplementary Figure 2B**). As for the conservative regions, a good structural match was found in the peptide sequence motif CGSCWAFS, cathepsin L signature sequences GCNGG, ERFNIN, GNFD and the Gln^118^, Cys^124^, His^260^, Asn^281^ active site residues (**Supplementary Figure 2C**).

#### Cathepsin Z

The genome contig encoding *C. shasta* cathepsin Z was not complete. The partial sequence was 1,334 bp and two introns (26-27 bp) were observed. We identified four isoforms in the transcriptome with a similarity of 97-99% (27-57 bp differences, corresponding to 6 different positions and/or inserts) (**Supplementary Tables 3** and **4**). Inserts of 24 and 22 bp were observed in two of the four transcriptome isoforms, possibly representing two splice junction events, one of them in the CDS and the other in the 3’UTR region. The CDS was 861 bp over the 1869 bp transcript, and a 286 aa ORF was predicted. *C. shasta* cathepsin Z functional annotation indicated it belongs to the family C1 (papain family), with cysteine-type peptidase activity and involved in the lysosomal pathway. Primary organization of cathepsin Z (**Figure 2C** and **Supplementary Table 5**) revealed a signal peptide and a peptidase C1 domain, with four active sites (Gln^65^, Cys^74^, His^222^, Asn^247^), an active site mini loop (His^66^, Leu^67^, Pro^68^, Lys^69^, Tyr^70^) and five S2 subsites (Ser^116^-Ser^117^, Ser^196^, Asn^220^, Glu^223^ and Ile^279^). *C. shasta* cathepsin Z did not contain the two highly conserved ERFNIN and GNFD motifs that are characteristic for the pro-regions of cathepsin L-like enzymes (Turk et al., 2012).

The 3D model of *C. shasta* cathepsin Z (**Figure 2C**) was constructed based on six templates sharing 31-40% identity with the target molecule. 100% of residues were modelled at 100% confidence, one residue at the C-terminus was modelled *ab initio* (**Supplementary Table 6**). The *C. shasta* cathepsin Z model stereo-chemical quality analysis positioned 79% of residues in the most favored regions (**Supplementary Figure 3A**). The comparison of the secondary structure alignment and the superposition of the 3D structures of *C. shasta* cathepsin Z zymogen and human procathepsin X (PDB: 1DEU) showed a relatively complete structural overlap in the central part of both molecules while the terminal parts of *C. shasta* protein, especially the N-terminus, showed a substantial level of structural deviation from the superposed molecule, which was mainly caused by a different composition of amino acid residues (**Supplementary Figure 3B**). The conserved regions (CGSCWAFS, GCNGG) and the the catalytic site residues (Gln^65^, Cys^74^, His^222^, Asn^247^) structurally overlapped in both molecules (**Supplementary Figure 3B**). The structural difference was observed in the active site mini-loop that is a unique feature of cathepsin X/Z molecules. This loop typically includes a short three-residue insertion that protrudes into the active site. In human cathepsin X, the Tyr on the C-terminal side of the loop forms the surface of the S1 subsite, and the His on the N-terminal side modulates both carboxymono- and carboxydi-peptidase activities of human cathepsin X. In human cathepsin X, the mini-loop is formed by a HIPQY motif while the corresponding *C. shasta* cathepsin Z region is represented by HLPKY forming a coil and a short α-helix (**Supplementary Figure 3C**).

#### Aminopeptidase-N

The genomic sequence of *C. shasta* aminopeptidase was 3,296 bp and contained 17 introns (21-28 bp). Two isoforms were assembled with 99% similarity and a 21 bp insert in the 5’UTR region, which may suggest a splice junction in the 5’UTR region (**Supplementary Tables 3** and **4**). 2,567/2,904 bp corresponded to CDS, and an 855 aa ORF was predicted (both isoforms were predicted as the same protein). *C. shasta* aminopeptidase-N functional annotation showed it belongs to family M1 with a metallopeptidase activity and zinc-ion binding; KEGG annotation suggested involvement in a system of regulation of fluids and electrolytes balance (**Table 1**). Basic primary organization of *C. shasta* aminopeptidase predicted protein (**Figure 2D** and **Supplementary Table 5**) indicates an M1 peptidase domain, with up to 9 active sites (His^90^, Glu^92^, Gly^228^-Ala^229^-Met^230^-Glu^231^, His^264^-Glu^265^, His^268^, Glu^287^, Asn^291^, Phe^345^, Tyr^350^). Three of these sites were also annotated as zinc binding sites (His^264^, His^268^, Glu^287^). Towards the C-terminus, an ERAP1-like C-terminal domain was predicted that is represented by 16 α-helices organized in 8 HEAT-like repeats forming a concave face towards the active site of the peptidase.

The 3D model of *C. shasta* aminopeptidase (**Figure 2D**) was constructed based on six templates sharing 25-29% identity with the target molecule. 98% of residues were modelled at 100% confidence, 21 residues at the N- and C-terminus were modelled *ab initio* (**Supplementary Table 6**). The stereo-chemical quality analysis of *C. shasta* aminopeptidase model showed 90% of the residues positioned in the most favored region (**Supplementary Figure 4A**). The comparison of the secondary structure alignment and the superposition of the 3D structures of *C. shasta* cathepsin aminopeptidase and human aminopeptidase N (PDB: 4FYT) showed a relevant structural overlap in the central part of both molecules, while the terminal parts of *C. shasta* protein were much shorter compared to the superposed molecule due to the lack of 119 N-terminal and 15 C-terminal amino acid residues (**Supplementary Figure 4B**). Identical structural overlap was found in the conserved HEXXHX_18_E zinc-binding and GAMEN catalytic motifs (**Supplementary Figure 4C**).

#### Stefin

The genomic sequence of *C. shasta* stefin was 447 bp and contained 2 introns (20-23bp), with only one isoform assembled from the transcriptome (**Supplementary Tables 3** and **4**). 285/434 bp corresponded to CDS, and a 94 aa ORF was predicted. *C. shasta* stefin was poor on functional annotations by different databases although the molecule clearly contained a cystatin-like domain belonging to the I25 family, primarily with inhibitory activity on papain-like cysteine peptidases of family C1 (**Table 1**). Basic primary organization of the predicted stefin (**Figure 2E** and **Supplementary Table 5**) indicated a cystatin domain with typical stefin motifs including the N-terminal Gly^5^, the central GXVXG motif (Gln^49^, Val^50^, Val^51^, Ala^52^, Gly^53^) and the LP pair (Leu^76^, Pro^77^).

The 3D model of *C. shasta* stefin (**Figure 2E**) was constructed based on two templates sharing 22-27% identity with the target molecule. 96% of residues were modelled at 100% confidence, 4 residues at the N- and C-terminus, were modelled *ab initio* (**Supplementary Table 6**). The *C. shasta* stefin model showed up to 86% of the residues positioned in the most favored region, according to the stereo-chemical quality analysis (**Supplementary Figure 5A**). The comparison of the secondary structure alignment and the superposition of the 3D structures of *C. shasta* and *Clonorchis sinensis* (PDB: 5ZC1) stefins showed that both proteins are very similar in their secondary and tertiary structures. The exceptions were the N- and C- terminals that, in *C. shasta* protein, were much shorter compared to the superposed molecule due to the lack of 36 N-terminal and 9 C-terminal amino acid residues (**Supplementary Figure 5B**). A strong structural overlap was found in the conserved stefin regions (N-terminal glycine residue, QXVXG and LP) of both molecules (**Supplementary Figure 5C**).

All characterization and functional annotations for each molecule are summarized in **Table 1** and **Supplementary Tables 3**–**5**. Templates and scores for all protein models calculated in Phyre2 are reported in **Supplementary Table 6**.

### Homologous Proteases in Myxozoans Transcriptomes

Cathepsin L homologs were the most abundant proteases found in the myxozoan transcriptomes mined (71 sequences), followed by aminopeptidase homologs (39). *M. squamalis* showed the largest repertoire of cathepsin L homologs (18) in stark contrast with the closest related species *H. salminicola* (2). *M. cerebralis* had the largest set of aminopeptidase homologs (12) followed by *T. bryosalmonae* (9). All myxozoan transcriptomes had at least one homologous sequence to each of *C. shasta* proteases (**Table 2**) except *K. iwatai* (missing cathepsin D and Z) and *S. molnari* (missing cathepsin Z). *C. shasta* cathepsin L sequence matched previously characterized proteases in *T. bryosalmonae* cathepsin L-like (contig_2419) and cathepsin L2 (contig_6351) (Faber et al., 2021), and in *S. molnari* cathepsin L1 (Smolnari_BS_DN24597_c0_g1_i1), cathepsin L2 (Smolnari_BS_DN24597_c0_g2_i1) and cathepsin L3 (Smolnari_BS_DN14729_c0_g1_i1) (Hartigan et al., 2020; See **Supplementary File 2**).

## Discussion

**Figure 3:**
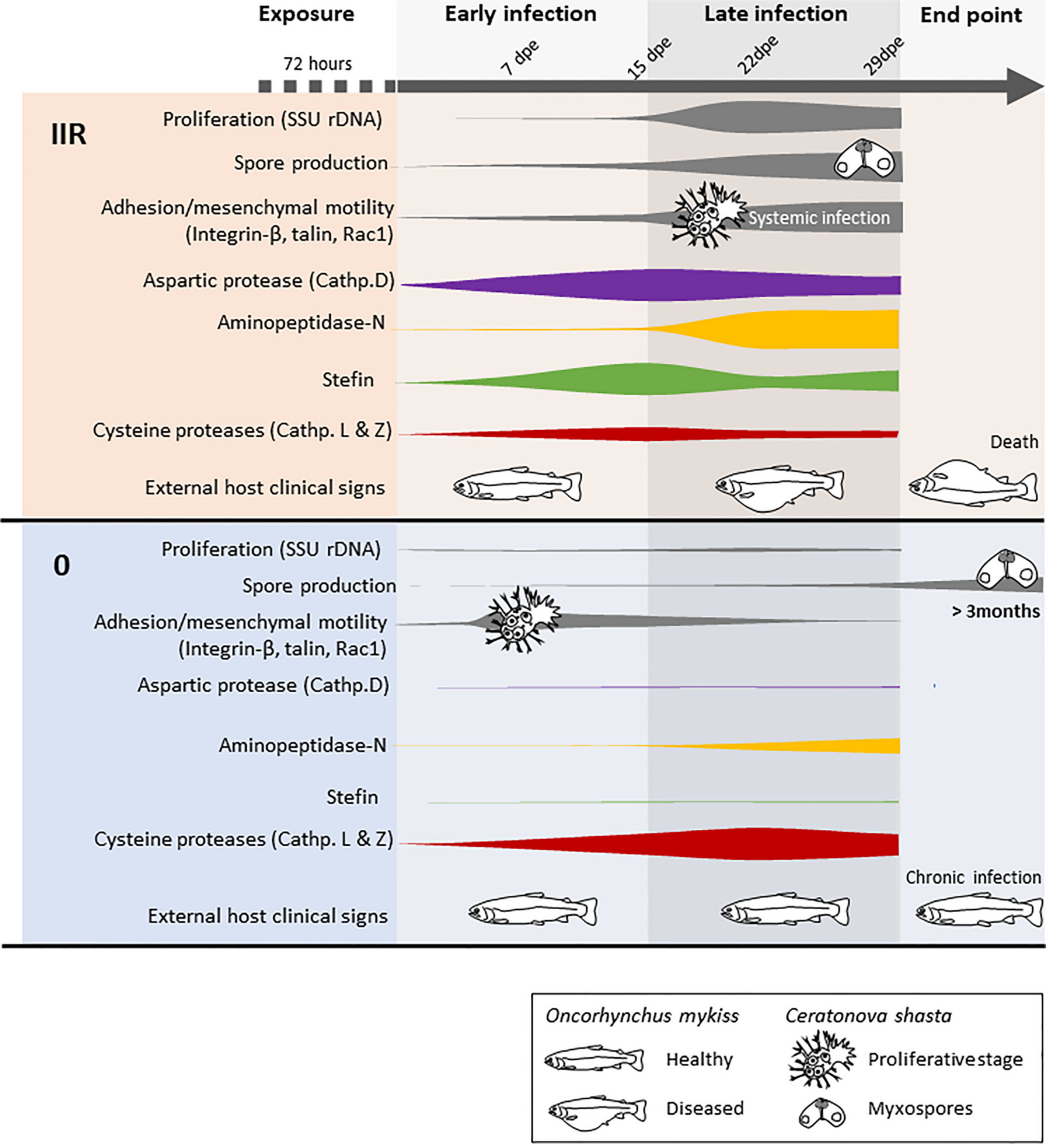
Model summarizing the infection dynamics and proliferation of *Ceratonova shasta* genotypes IIR and 0 in the intestine of rainbow trout *Oncorhynchus mykiss*, combining results from visual observations, parasite molecular quantification, motility and host clinical signs (modified from Alama-Bermejo et al., 2019) with proteases and stefin gene expression.

The differential expression of four *Ceratonova shasta* proteases and one stefin and the *in silico* characterization and annotation of these molecules provides a first comprehensive overview of their protein characteristics and the differential uses of these enzymes between parasite genotypes with differing virulence (**Figure 3**). We consider these as potential candidates for developing new chemotherapeutic strategies against *C. shasta* specifically, and possibly against myxozoans in general.

### A Boost of Aspartic Protease Expression Preceeds Massive Proliferation of the Virulent *C. shasta* Genotype

The *C. shasta* protease with a functional annotation of “aspartic protease (cathepsin D)” (i.e. a lysosomal protease) had structural similarities to pepsinogen, a major enzyme of gastric juice, which suggest that *C. shasta* cathepsin D is involved in host exploitation after protein digestion, similar to other parasites (Sojka et al., 2016). This protease is significantly upregulated only in the virulent genotype IIR, beginning early in the infection and with a peak of expression at 15 dpe, when IIR infection is established in the intestine and proliferation, sporogenesis and systemic infection is increasing (**Figure 3**). *C. shasta* aspartic protease is present in proteomic data from developmental stages but nearly absent in myxospore proteomes (Brekhman et al., 2021). We assume that *C. shasta* cathepsin D could contribute to the rapid proliferation and virulence of this genotype by causing the destruction of the intestinal extracellular matrix in order to satisfy the high demand for nutrients by the developmental stages. This cathepsin’s role in *C. shasta* virulence would thus be in line with the ¨eating¨ function (Cassone et al., 2016) of this class of proteases in other parasites, like blood flukes, malaria and ticks, where cathepsin Ds are mostly involved in host hemoglobin digestion (Coombs et al., 2001; Sojka et al., 2016). However, aspartic proteases can have broad biological functions, including so-called ¨heating¨, which involves triggering inflammation in the host (Cassone et al., 2016). We observed that genotype IIR infection results in upregulation of the pro-inflammatory cytokines IL-6 and IL-8, especially IL-6 at 15 dpe in rainbow trout (Taggart-Murphy et al., 2021). This mechanism is considered as a virulence-associated trait in the eukaryotic pathogen *Candida albicans*, in which secretory aspartic proteases contribute to the pathogenesis of inflammatory mucosal lesions by inducing upregulation of pro-inflammatory cytokines (Schaller et al., 2005). Further studies involving recombinant proteins should focus on determining the functions of *C. shasta* cathepsin D and potential effects of this protease on the host inflammatory response.

### The Less Virulent Genotype May Use Cysteine Proteases to Evade the Host Immune System

Elevated cysteine protease expression is commonly associated with virulent or pathogenic parasite species, and is reduced or even absent in closely related non-pathogenic species e.g. *Entamoeba* spp. (Bruchhaus et al., 2003; Ximénez et al., 2017). Intriguingly, both papain-like cysteine proteases we identified in this study were upregulated only in the low virulent genotype 0. Upregulation occured at all timepoints and especially when the infection proliferated in the intestine (**Figure 3**) and both proteases seem to be related to lysosomal pathways. Genotype 0 infection in rainbow trout shows a cellular immune response with little tissue damage. The reduced upregulation of both inflammatory and regulatory cytokines in the host suggests that type 0 modulates the inflammatory response (Taggart-Murphy et al., 2021). Parasite-derived cysteine proteases could contribute to neutralizing immune pathways (Bird et al., 2009) by degrading host antibodies or complement proteins. *Fasciola hepatica* cathepsin L, which showed similar structure to *C. shasta* cathepsin L, can cleave host immunoglobulins, preventing the attachment of eosinophils to the parasite surface (Carmona et al., 1993). *Trypanosoma brucei brucei* cathepsin L contributes to the parasite’s ability to resist lysis by human serum (Alsford et al., 2014), and its inhibition results in trypanocidal activity (Steverding et al., 2020). In some cases, cysteine proteases may alter the host immune response by inducing a T helper 2-type (TH2) response instead of T helper 1-type (TH1), which can be less effective against certain pathogens (Giordanengo et al., 2002). In a recent transcriptomic study of susceptible rainbow trout to genotype IIR, signature molecules of TH1 and TH2 response were upregulated (Barrett and Bartholomew, 2021); however, these responses are not known for type 0 infected fish. A full profile on the immune response of rainbow trout to genotype 0 could provide further valuable insights on disease control.

Interestingly, functional annotation suggested that *C. shasta* cathepsin L has a role in antigen processing and presentation. Vertebrate cathepsins function in the antigen processing and presentation pathway of the adaptative immune response (Blum et al., 2013). Although it is currently accepted that invertebrates lack this type of response, in some cases invertebrates can develop a long-term protection against pathogens, which suggests that they may possess alternative pathways (Brown and Rodriguez-Lanetty, 2015). These authors proposed a model of molecular defense priming for the cnidarian *Exaiptasia pallida* which includes the use of cathepsins for activation of endosomal cell membrane TLRs (Toll-like receptors). However, it is unknown if myxozoans, as reduced cnidarians, possess this alternative pathway and if they could use it to evade the host immune system. In summary, we hypothesize that in genotype 0 infections, *C. shasta* cathepsin L aids in evading host immune responses and in preventing an acute inflammatory response, resulting in the chronic and long-lasting infections characterized by this low-virulence genotype, but the mechanisms used by the parasite to endure in the host are still unknown.

Homology matches suggested cathepsin L is a required protease in development of multiple myxozoan species. Interestingly, we observed that two closely related species, *M. squamalis* and *H. salminicola* have large differences in their repertoire of cathepsin L homologs, which could be in line with the observed gene loss and simplification of *H. salminicola* genome (Yahalomi et al., 2020). *C. shasta* cathepsin L showed high sequence similarity to previously characterized cathepsins L in *T. bryosalmonae* and *S. molnari* (Hartigan et al., 2020; Faber et al., 2021). None of these cathepsins showed significant differences in expression between different developmental stages and hosts, except for Sm_CL3 which had higher expression in sporogonic gill stages than in proliferative blood stages, which suggests a role during sporogenesis. Like *C. shasta* cathepsin L, all three *S. molnari* cathepsins share high structural similarity to *F. hepatica* cathepsin L. Sm_CL1 and CL2 also possess a signal peptide, and may be involved in blood feeding (Hartigan et al., 2020). All these results suggest different but essential roles of cathepsin L proteases in myxozoan life cycles and development, making them important candidates for further research.

*C. shasta* cathepsin Z belongs to a group of papain-like lysosomal cysteine peptidases whose biological functions are poorly known. Our annotations showed similarity of the peptidase unit to cathepsin Z of another myxozoan, *M. cerebralis*. This protein is highly transcribed in trout cartilaginous tissues, like the cranium and gills, after 24 dpe and during sporogenesis at 54 dpe (Kelley et al., 2003). While this cathepsin in *C. shasta* could have a histolytic function, we did not observe elevated expression in the more virulent genotype IIR, instead we observed high expression only in genotype 0 infection, where the tissues remain mostly intact. As the functional annotations of this protease also suggest its involvement in lysosomal pathways, we believe this protease could have similar roles to cathepsin L.

The patterns of expression observed for these cysteine proteases do not exclude the possibility that other cysteine proteases could have important roles in pathogenesis of genotype IIR. The protease repertoire of *C. shasta* includes other cysteine proteases (**Table 2**; Alama-Bermejo et al., 2020; Ahmad et al., 2021), which may contribute to the parasite’s proteolytic activity on the host intestine extracellular matrix, as reported in other histolytical parasites e.g. *Entamoeba histolytica* (Kissoon-Singh et al., 2011), where cysteine proteases are primary virulence factors.

### Aminopeptidase-N Assists *C. shasta* Nematocyst Formation

Inhibition of aminopeptidases has been shown to negatively affect the development and survival of parasites, and could be an important therapeutic strategy using small-molecule inhibitors, or to help define vaccine targets (Drinkwater et al., 2017). Aminopeptidases belonging to the M1 family have special relevance in apicomplexan parasite life cycles. *Plasmodium falciparum* M1 aminopeptidases are known to be involved in the last steps of hemoglobin digestion in intraerythrocytic stages, releasing amino acids from small peptides (Dalal and Klemba, 2007). An aminopeptidase-N is involved in the development of *Eimeria tenella* during oocyst sporulation (Gras et al., 2014). *C. shasta* aminopeptidase-N was expressed mostly at latter time points in both genotypes, and was coincident with the onset of spore production (**Figure 3**). These expression results align with the proteomic data of the type IIR nematocyst, where the aminopeptidase-N protein was found to be of special significance (Piriatinskiy et al., 2017; Brekhman et al., 2021). These findings and the role of aminopeptidases in other parasite groups suggest that this zinc-metalloprotease could be involved in the formation of nematocysts during *C. shasta* sporogenesis, more specifically in the pressurization of nematocysts. According to its functional annotation, *C. shasta* aminopeptidase is involved in the renin-angiotensin system, a hormone-enzymatic system present in invertebrates and vertebrates. This system is implicated, amongst others functions, in osmoregulation (Salzet et al., 2001). Nematocysts of cnidarians, and the homologous polar capsules of myxozoans, are highly pressurized cell compartments and thus aminopeptidase-N could have a role in regulating fluid and electrolyte balance in the formation of this structure in *C. shasta.* Regulation of osmotic potential is the driving force in the process of tubule elongation during discharge, which is the first step of parasite invasion (Americus et al., 2020). The abundance of aminopeptidase homologs in myxozoan transcriptomes supports a common role of these enzymes in their development. In fact, aminopeptidase homologs were most abundant in transcriptomes of *M. cerebralis* actinospores and *T. bryosalmonae* spore sacs (**Table 2**), which suggests additional roles for these proteases during actinospore/malacospore formation. Further transcriptomics comparisons between vertebrate and invertebrate stages of myxozoans are needed to fully elucidate the role of aminopeptidases in the parasite development.

### The Virulent Genotype Requires Endogenous Cysteine Protease Regulation by Its Stefin

Stefins are tight binding intracellular inhibitors of the papain family of cysteine proteases that generally lack a signal peptide, and hence are usually involved in regulation of endopeptidases (Turk et al., 2008). Myxozoan stefins are atypical molecules with a chimeric structure combining motifs of type 1 cystatin or classical stefins and type 2 cystatins (Bartošová-Sojková et al., 2021). While some myxozoan stefins posses a signal peptide, *C. shasta* stefin lacks this feature, which suggests it has intracellular/cytoplasmic localization. As observed in other parasites (e.g. Liu et al., 2019), this suggests that the *C. shasta* stefin could be regulating its own cysteine proteases and, to a lesser extent, host cysteine proteases. The contrasting stefin expression between genotypes suggests different demands on cell regulation due to different life strategies. Genotype IIR shows higher expression of stefin, with a particularly high fold change at 15 dpe, probably to regulate parasite cysteine proteases used for rapid proliferation and spore formation (**Figure 3**). In fact, this stefin is present in IIR *C. shasta* proteomic data from ascites but absent from mature myxospores (Brekhman et al., 2021). Similarly, other parasite stefins are expressed in developmental stages or trophozoites during encystation, when large volumes of intracellular components are required and protection from cysteine protease activities is needed to avoid intracellular damage (Lee et al., 2013).

Host cysteine proteases have essential roles in activation and development of immune responses in pathogen recognition and elimination (Kopitar-Jerala, 2012). Recently, abundant rainbow trout cysteine proteases were observed to be upregulated in the intestine of fish exposed to genotype IIR at 14 and 21 dpe (Barrett and Bartholomew, 2021). *C. shasta* could be responding to these proteases by using inhibitors. Stefins can be major released antigens in protozoans and trematodes, suggesting extracellular functions in controlling host cysteine proteases and/or with immunomodulatory effects (Tarasuk et al., 2009; Liu et al., 2019). If secreted, even by non-canonical pathways, *C. shasta* stefin could neutralize host cysteine proteases released to eliminate the pathogen. Secretomic analyses should be implemented to investigate which molecules are present in the interplay between proliferative stages and host cells.

## Conclusions

*Ceratonova shasta* genotypes have different life strategies and we have now shown that two genotypes, one more virulent than the other, have different expression of key genes. These observations lead us to propose that proteases are important components of host-parasite interactions in myxozoans, and are essential mediators of parasite virulence. We suggest that *C. shasta* aspartic protease and stefin are key for the rapid proliferation and metabolism of genotype IIR and thus should be considered candidates for drug and/or vaccine development, as in other parasite models (Sojka et al., 2016; Ranasinghe and McManus, 2017; Dong et al., 2018). Other proteases, like cathepsins L and Z, are more likely implemented in parasite immune evasion, and could explain the lack of acute inflammatory response in the low virulent type. In contrast, aminopeptidase-N is probably fundamental for spore formation, independent of the virulence level of the genotype, rendering this protease as a universal target.

3D modeling showed that *C. shasta* proteins possess similar folds and biochemical characteristics as the superposed molecules. However, wet-lab experimental work is essential to validate the predicted structures of these *C. shasta* proteins, to characterize their exact functions and explore them as novel drug targets applicable for the development of new therapeutic strategies for myxozoan pathogens in aquaculture systems.

## Data Availability Statement

The datasets presented in this study can be found in online repositories. The names of the repository/repositories and accession number(s) can be found in the article/**Supplementary Material**.

## Ethics Statement

The animal study was reviewed and approved by Oregon State University Institutional Animal Care and Use Committee under approval ACUP #4666.

## Author Contributions

GA-B, AH, and JB conceived and designed the study. GA-B, SA, and PB-S collected, generated, and analyzed the data. AH and JB supervised and GA-B wrote the manuscript. All authors provided critical feedback and helped shape the research, analyses and manuscript.

## Funding

GA-B, AH, and wet lab work was funded by the Czech Science Foundation EXPRO grant #19-28399X (AQUAPARA-OMICS; 2019-2023). PB-S was funded by the Ministry of Education, Youth, and Sports of the Czech Republic grant # LTAUSA17201 and the Czech Science Foundation grant # 21-16565S. JB and SA were funded by the Bureau of Reclamation, U.S. Department of Interior through Interagency Agreement #R19PG00027, as part of its mission to manage, develop, and protect water and related resources in an environmentally and economically sound manner in the interest of the American public. The funders had no role in study design, data collection and analysis, decision to publish, or preparation of the manuscript. Mention of trade names does not imply U.S. Government endorsement.

## Author Disclaimer

The views in this report are the authors’ and do not necessarily represent the views of Bureau of Reclamation.

## Conflict of Interest

The authors declare that the research was conducted in the absence of any commercial or financial relationships that could be construed as a potential conflict of interest.

## Publisher’s Note

All claims expressed in this article are solely those of the authors and do not necessarily represent those of their affiliated organizations, or those of the publisher, the editors and the reviewers. Any product that may be evaluated in this article, or claim that may be made by its manufacturer, is not guaranteed or endorsed by the publisher.

## Supplementary Material

Supplementary Figure 1
*Ceratonova shasta* IIR cathepsin D. **(A)** Stereo-chemical quality analysis: Ramachandran plot and statistics using PROCHECK; **(B)** Superposition of *C. shasta* cathepsin D and tick *Ixodes ricinus* cathepsin D zymogen (PDB: 5N7N) and **(C)** Structural alignment of *C. shasta* cathepsin D and tick cathepsin D (PDB: 5N7N) using Phyre2 modelling and UCSF Chimera for visualization (in red: aspartic catalytic sites).

Supplementary Figure 2
*Ceratonova shasta* IIR cathepsin L. **(A)** Stereo-chemical quality analysis: Ramachandran plot and statistics using PROCHECK; **(B)** Superposition of *C. shasta* cathepsin L and *Fasciola hepatica* procathepsin L (PDB: 2O6X) and **(C)** Structural alignment of *C. shasta* cathepsin L and *F. hepatica* procathepsin L (PDB: 2O6X) using Phyre2 modelling and UCSF Chimera for visualization (in red: catalytic sites; asterisks: conserved residues in the corresponding ERFNIN and GNFD motifs).

Supplementary Figure 3
*Ceratonova shasta* IIR cathepsin Z. **(A)** Stereo-chemical quality analysis: Ramachandran plot and statistics using PROCHECK; **(B)** Superposition of *C. shasta* cathepsin Z and *H. sapiens* procathepsin X (PDB: 1DEU) and **(C)** Structural alignment of *C. shasta* cathepsin Z and *H.sapiens* procathepsin X (PDB: 1DEU) using Phyre2 modelling and UCSF Chimera for visualization (in red: catalytic sites).

Supplementary Figure 4
*Ceratonova shasta* IIR aminopeptidase-N. **(A)** Stereo-chemical quality analysis: Ramachandran plot and statistics using PROCHECK; **(B)** Superposition of *C. shasta* aminopeptidase-N and *H. sapiens* aminopeptidase-N (PDB: 4FYT) and **(C)** Structural alignment of *C. shasta* aminopeptidase-N and *H. sapiens* aminopeptidase-N (PDB: 4FYT) using Phyre2 modelling and UCSF Chimera for visualization (in red: active sites, in yellow: Glutamyl residue required for catalytic activity, asterisks: active sites also annotated as zinc binding sites).

Supplementary Figure 5
*Ceratonova shasta* IIR stefin. **(A)** Stereo-chemical quality analysis: Ramachandran plot and statistics using PROCHECK; **(B)** Superposition of *C. shasta* stefin and *Clonorchis sinensis* stefin (PDB: 5ZC1) and **(C)** Structural alignment of *C. shasta* stefin and *C. sinensis* stefin (PDB: 5ZC1) using Phyre2 modelling and UCSF Chimera for visualization.

Supplementary File 1
*Ceratonova shasta* proteases and stefin transcriptomic contigs (including isoforms) and predicted protein sequences.

Supplementary File 2 Homology search results: homologous predicted protein sequences from the myxozoan transcriptomes mined and their MEROPS annotation. Predicted protein sequences are included in the file.

Supplementary Table 1 Proteases and stefin genes relative change mean ± SD using 2^-ΔCq^ method per gene, sampling day post exposure (dpe) per each genotype (0 and IIR) against their respective reference genes in the intestine (n = 5 fish per genotype, otherwise indicated in the table). Statistical differences between mean fold changes at different time points (7, 15, 22, 29 dpe) were calculated using Tukey´s method for multiple comparison after one-way ANOVA or t-test for normally distributed data. For non-normally distributed data, Kruskal-Wallis with Dunn’s multiple comparison was applied. Letters in parentheses indicate significant difference groupings.

Supplementary Table 2 Proteases and stefin genes fold change mean ± SD calculated using 2^-ΔΔCq^ method per gene and sampling days post exposure (dpe) between *Ceratonova shasta* genotypes IIR and 0 in the intestine (n = 5 fish per genotype, otherwise indicated in the table). Statistical differences between mean fold changes at different time points (7, 15, 22, 29 dpe) were calculated using Tukey´s method for multiple comparison after one-way ANOVA or t-test for normally distributed data. For non-normally distributed data, Kruskal-Wallis with Dunn’s multiple comparison was applied. Letters in parentheses indicate significant difference groupings.

Supplementary Table 3 Genomic evidence (length, position in contig, number of introns and their position) for *Ceratonova shasta* proteases and stefin.

Supplementary Table 4
*Ceratonova shasta* proteases and stefin genes and isoforms found in the transcriptome assemblies, % of similarities, differences at the RNA and predicted ORF and potential relevance of these changes.

Supplementary Table 5 Predicted domains, sites and conserved motifs annotated from a combination of databases (Uniprot, NCBI nr, CDD, SMART, Prosite, InterProScan) using predicted protein sequence obtained with ORFfinder (NCBI). Graphical representation can be found in Figure 2.

Supplementary Table 6 List of templates used to model the final 3D structures of *Ceratonova shasta* target proteins using Phyre2. The template structures used for superposition with *C. shasta* proteins are grey-shaded.
