# Supplementary material for: Proteases as Therapeutic Targets Against the Parasitic Cnidarian Ceratonova shasta: Characterization of Molecules Key to Parasite Virulence In Salmonid Hosts
Source: Front Cell Infect Microbiol. 2022 Jan 7;11:804864. doi: 10.3389/fcimb.2021.804864 (PMC8777295; doi:10.3389/fcimb.2021.804864)
Supplement: Supplementary Figure 1 — Ceratonova shasta IIR cathepsin D. (A) Stereo-chemical quality analysis: Ramachandran plot and statistics using PROCHECK; (B) Superposition of C. shasta cathepsin D and tick Ixodes ricinus cathepsin D zymogen (PDB: 5N7N) and (C) Structural alignment of C. shasta cathepsin D and tick cathepsin D (PDB: 5N7N) using Phyre2 modelling and UCSF Chimera for visualization (in red: aspartic catalytic sites). [file DataSheet_1.pdf]

```
>TRINITY_DN7023_c0_g1_i1 len=1266 path=[2604:0-1132 2605:1133-1168 2606:1169-1265]
[-1, 2604, 2605, 2606, -2] Match_Acc=Q76EJ2 Gene=Cathepsin_D_
AAAAATAAAGTTTTTATTTGAAGAGTCTTGTCCATTTGGGTAGTCTATAGGATGTATGTT
GTGGATATATTTTTAATATATCAACTTAAAAAAATTAAAATGCTTTAAAAGCATAGTCAA
TTAATAATTTTTGACTGTTCCAAATGAAACTGTTTGGTTTTCAATATCAAATATGGTATA
ATATTTTCCCATAAAAACATCACCTAAAATCCATGAAATACCTGATGAAGACGGTAAATC
TATTCCCATAAAAGCACTTAAACATACTTCCGATTCTTGTATAACATAATCATTTGGATA
TAATGTCATTATGACATTATTTATCTTAAAATCTACATTTGGTAAGTGAGGAATATCTTC
GCATTTAAAAATACTAGCATTGTTGAATAATTTGTGTGCTCCCAATTTTTTATTCAAAAT
TTCTATGTCATGAAGAGGTCCAGCAATTAAAGAAGTCCCACTATCAACTGCAGCCATGCA
TCCATCAATACAAATGTTCGTTACATCACTTGTAATTGCATCAACTTTAATAGTCCAATA
TAACGGGGTTACAACTTTTGTTGTAACCAAAATTTCCCCCAACTTATCATAATCAACACC
TCCAAACATTATTTCTCCTCCAGTAGGGTCTTCGGTATCTCTATCTATATAAAATGAAAA
TTTTGGTTGAATTTCAGGGTTTTGTTTCAAAATAGTGTTAAATACTGGACTAGCTTTGAA
AGCGCTTAAGCCAGGAAAACCTAAACCAAGAATACCATCAAATTTAGCTAATCCAAATGG
TACAAATGGCATATCAGTATTTTCACCAAAAGTCTGTTTAGTTGCTTTCATACCAGCAAA
ATTTATATCATCATAGGATAGTATACCTGCTACGGTTCCTGAACCATAAGATAATTGATA
ATGATTGCCGTTTTCTATATATGTAGAGGATTTTTTATAGTTATATTTGGGATGTAATTT
ACAAGCTAAACTGCTCAATTTACATCTTTTAGATGGAACCCAAAAATCAGAAGAACCTGT
GTCAAAAACAACCGTAAAATTTTGTACGGGAGTACCTAATGATATTACTCCATAATATTG
AGCATCCTTAAAATTTAGTAAATCTTCTTTTCCTGGATCAGCACTGTTTTTTTGAACAAG
ATTAAGCTTATCCAAAAAATGTATAAGGGATCTTTCTTGTTTATGTAGTTTAACTTTGAA
AGATTTTGAACAATTAGAAAGACATACAAAAGATATTACTAAAACCAAAATTAACATTTT
ATATAT
>TRINITY_DN22765_c0_g1_i2 len=2904 path=[6278:0-801 @6286@!:802-2646 6283:2647-2690
6284:2691-2903] [-1, 6278, 6286, 6283, 6284, -2]
TATATCACATAAAAATTTAATTTAGTTCTAACAACCCTATACTACATAATCCATATATTG
CCCACCCAATAAACTAATTTCAAATATTTGCACATGTTTTTAATAAATCTTCAGGTAATT
GTTTCTTAAAATATGCTGATATCCAAGTGGAAGCTTGACATTTTCTTTTTATTGCTAATG
TTGTATTATTTCCAGTTTTTTTTTCATTCAATTTTTCCATAACCATGTTTTTCACAATAG
AATTCTCATTTATTAACAGTGTATTTAGAAAAATTTCAATTTGTTGCAGAGTCTCTTTGT
CTGACGTTAATGTAACAACTTTTAACATAAATTCAAGCATAACACTTTTTTTCATTATAA
ATGAAAGATCATTATAAGATTCTTTTAGCTTGGTAAAGCAATTCATTTTGTCAGAGCAAA
ATTTGTAAATTAGAATTAAAAATGAAAGCTTGTCCTGGATTTTTACGTTTTCTGAAGTTG
TTACTCTTAATATATTTTGTTGTGAAAATTCTCCTTCATTAAATGAAGCTATAACTTTCA
TCAAAAACTTTCTATCATAATCGCTTGTTGAATTCCTGTACAAGTTCCACAATTTAGCCA
AGATTGTGGCGTTCCGCTTGTCAACTCCATACAAAATGGCCAATGTTCTTATTTCCTTAT
ACTTAAATCCAGGTAAATTTTTATCCATTAAACGTTGCAACAACTTTTGCAAAGAATCTT
TCAAATCTGGAAGAGTTCCGTATTTAAGAGCAAAAAGAGCTACTTCATACTGTTGTAGAG
TTTCTAATGGATTTGATTCTTTAAGAAGAGTATCTATGTTTATCAAATGTTCTTGAAGTC
TCCATAATAAAATCTTGTATAGCTTAATAATAGATTTATGCTCAATAACATCAAATAAGC
AAGAATATTTTGACCTCATCATAGTCCAAGGTAAATAATCATTTTCATTTTGTAAGTATA
AGATCAAGTTGAAGACAATTTCTGGTGATATCATTCTGTCACAAGCTAATTTGAATGCAT
CATGGATTAATCCAGATCTATCTATTGGGGTCAAAACATTATGATTTTCTTTTAAGACAC
TTGAGATGTTTTTCCATCCTTGTTCATCATAATTTACAATATAAAATCCACTCATCCCAG
CATTAACTTTGAACCATTTGACGTTTTTTGATAAGATAATTTCTGCAGGCGAAATTGGTG
TCAATAATATTGTTCTTACATTCAAGGGAGATTCACTATCGATCACATTGACTGGTATAT
ACCATAAATCGTTTGAAAAATTATTTAAAGTATTTTCATTTTCAATGTCCTTCCCATTCA
AAGCGATATAAGAGTGTTGGAATAAAGTTATCACAACTCTATTTTCTTCATTTTTTACGG
TTTTCATAGTTATCAATGGAAACCCTTTCTTGCTTACCCAATTATTCATAATCCCATTTA
```

TGTTTATATTCACAGTATATGTGCTTAAAATATTCCACAAGTTTTCACTTGTAGCAGTTT
TATATTTGTAAGTTTCTACATAATCTCGAATTCCTGCAGTAAAATTAGCGTCACCCATAA
AAGAATGAATCATTCTTAAAATAGCGGCTCCTTTGCTATATGAAATAGGATCAAAGAGTT
CTTCAATGTTTGTTGAAGTGCTTATTTGATGAGAAATTGCATGTGTACTATTTATACTAT
CCATTTCTATTGCTGAATCCCAACCACTAACAACCTGCAAGTCCAACATTTTAAACCTAT
TATCAAAGTGATCAGCTCCTATTAGTTCAATGTAATTAGCAAACCCTTCATTAAGCCAAA
TATTGTCCCACCAATCTAATGTAACGAGGTTTCCAAACCATTGGTGAGCCAATTCATGAG
AAATGACGGATATTATATCTTTTTTGGTGTCTATTGCAGTTCTTTTGTCATCATAAATAA
TATAAATATTTCTAAAAGTCAAAAGTCCCCAGTTTTCCATAGCACCACTTGGAAATTCTG
GTAATGCAATCAAATCAATTTTTTTGTATGGGTATTTTTGGTTAAATAGTTTTTCAAAGA
ATGGTAATATTTTTAAGGTTACGTTTCCAGCGAAATCTAGTTCCTCTATTCTGTTCGCAT
AGGATGGTGCATAAATATTTATTGAAATATCATTGACTGTGACTTTAGATTGTGAAATAA
AATCCCCAATAGAAAATGCTAAGAGATAGGTACTCATTTTGACTGTTTTTTCAAATATAG
TTAAAGAAATTTGGTTGGACAAATTACTTGTTTTTAAAATTTGAGTGTTTGCTATTACTC
TAGTTTTTGAAGGATGCTGGAAAAATAAAGAAAAAGTGGCTTTGAATGATGGCTCGTCAA
AACAGGGGAAAACAGATCTAGCGCCAATCGGCTCAAAATGAGTAGCCAACATAAATTTTT
TTGTATTATTTTGTTGGTAAAATATTTTGTACAACCCTTCTATCGTGGAAGAGTATTTTG
CTGTATATGAGATTTGTAGTAAGTATACATTTTTGGTTGACAACTGTGTATTTAGGTTAA
ACGTAACAATTTGATTATCATTATCGTAAACATAATTTACAATTTCAACGATATCATTTA
ATATAGTAGTTTCAATAGTCGATTGTATTTCATTACTGATATATGAGACTTTGTTGAAAA
TACCCATCTTAAAATATTATTATCTTACATTATTGTTGATTGTCAATGTAAATGATTTGA
TATTGATATGAGAGCTAATATGAAGGGTAATTTTAGTTACAGGTTCAAAAACGTTTAAAA
ATATGGTAACATTACCACTAAAATAGTTTTGGTTATTTCCAAGGTTTAGTTGAAGGTCAT
AATGTGATGGTAAAATGGTTTTATTTAATCTGTATATTGATTGAGAAATTTGATTTGAAC
ACAAAAATAATATTAAAATTTGAG
>TRINITY_DN31847_c0_g1_i1 len=993 path=[1941:0-992] [-1, 1941, -2] Match_Acc=R4WQK9
Gene=Cathepsin_L_
GATCTTTTTTGAATTAAAACAAATAAAAAGTTATAATTAAACTATCGGATAAACTGCCAT
TGTTGCAACACCACACATATTGTTTTTAAAACGAGCTATGTGCATATAGCCGTCAATTCC
CCAATTTGTACCCCAACTATTTCTCAATATATAATAATCGTGTTCTTTATGGTCTTTGCT
ATATCCAACCAAGACAACTGCATGATTCATGTCTTCTGTGCTGCAATATTTATCTTGATA
TATTCCTTTATCATAAAACTGGAAAGAAAATGGTGACGAATCAATTACAATAGAAACCGG
ACCATTGGTTTTAATAGCATGACTAAGAGCATATTCATTTTGAGTGATATTCTTGTATCC
CTTAACTTTAAAAAGAACTTTAGAATGATTATAATGACATTTATTGAATAAAGAAGTTTC
GTATGGATAATCCTCCCATGATTCAATCCCTTTTTTCTTCAAGTATTGAATAGCTAGGTA
AGGAAATCCACCATCACAACCAAAGTTAGAATGGTCACATGAAACTATTTGTTGAACACT
CAAGTTAAATAATTTTCCTTTTTTAATAGCGTATGCTGATTCGAGAGCACCAACCGTAGA
AAAAGCGTAACAAGATCCACATGAACCTTGAGATTTGATTGGGTTTACAACATTCTTATC
TCTCCAATCCACTTTATCAGATGAAGGTGCTTGTCTGTAATTGGACTTTGTTGGTTTGTA
TTCAACTTTCATTTTATTAGCCAAATTTTTCTTGTATTCCTCATTAGACAAGTGGCCGAA
TTGGTTAATTTTTAACGTAAAAGTCTTTGTGGGATCAGAATTGTGTTCTTCGATTTTTCT
TATATTTTCAAAAAATATTGAAAACCTCTTGGCATTTTCTTCTTTTGTAAATTCTAAGTT
GTATTTTGCCTTGTGTAATTCCCAAGGCTTGTCATACTGGCAGTTAACAAAAGAAAGCAA
TATTAAAATAAAGATGTTCATTAAAAGGTATTA
>TRINITY_DN55712_c3_g1_i4 len=1869 path=[5088:0-125 5089:126-149 5090:150-164
5127:165-267 5143:268-303 5134:304-348 5126:349-372 5141:373-379 5101:380-403
5133:404-744 5151:745-768 5154:769-789 5063:790-817 5150:818-924 5073:925-955
5147:956-1036 5152:1037-1057 5138:1058-1078 5131:1079-1117 5122:1118-1141
5149:1142-1202 5136:1203-1296 5130:1297-1374 5144:1375-1407 5135:1408-1463
5129:1464-1487 5153:1488-1502 5028:1503-1868] [-1, 5088, 5089, 5090, 5127, 5143,

5134, 5126, 5141, 5101, 5133, 5151, 5154, 5063, 5150, 5073, 5147, 5152, 5138, 5131, 5122, 5149, 5136, 5130, 5144, 5135, 5129, 5153, 5028, -2]
AATATTTACAAAATATCATTTAATTTATTTGCAGCAAACATTTTAATTTAATTAAAATAG
AAGATAATACAGTAGTACCTCGATCTACGCAATGGTATTCATAGCAGAATTCTGCTAGAA
CAATGAGCTGAGGTTCGAGTTACGAGATGGGAAAATTTTGAAGATTAATGATTTTGAATT
TACCGCCACATATGGCTAGTCGGTGCGTTGGTTGCTAGTTGGAGCATAACAATCTACTGT
TGTTTACAAATAATTAAAAGTAAACTAAAATTATAGTACAGTACAGTATCGTAACTGTGC
GATTCAAGTTTACCGCCGTCTCCTTGCCCCTCCCTTCTCCTCCAGCTCAGCCTTTGCTAC
CGTAGCTGATACAGTATCTAAACGTAAGTACCGACAGAATTTCCTGTTCAATGGCCACTC
TCTTCTTTTCTCATTCTATTTATTATTATTTATACTGCATTAATAGTTTGTATGAGAAA
AACATGCCTTTTTATGTTTAATACAGTGTTTTAGGGGAATTTTCAAGGGTAGGAACCAAT
TAAATTTTTTTCCCTTTATTTTTAATGAAAATAATTTGATTGACTTACGAGATGATCGAA
GAACGAGCTAGGTTATGTAACGAATAAATCTCGTGGGTCGATTTACTACTGTATATATAT
ATATTAAGAATTATTTGTCTATTAAAATCACAAAAAAATTAAAAAAACCTTATAGAATTT
TAGAATAAGATTGGAAAATCACATATTTCACTACCAATACCTAAAGAATAGTCTGCGTTC
GAATATTCATGGGTCGGAACTCTGAAAAATCCATCCTCACCCCAAAATTGTCCCCATGAA
TTTCTTACTATCCAATAAGGCAATGGTTTATTTTCTTCATCTACAGTCAGTCCCCAACCA
ACTATTGAAATTTCATGATCATTTCGTTCATTAGCTTTTTCCATATAAATTCCTCCTTTA
TAGTTTTTTAAAAAGTTATCGGATGCATACATTGAACAAGAAATTGGACCGTTTGTCATT
ATCTCCTTTTTCATCATGTTAGCAATCTTTACGATTCCAAACTCTTTAATAATCCATTTT
TTATAGTCTTTTACAGTGTAACATGGATCGTTAAAGTTAGGACATGTTGTGCACCTATTT
TCTTTATCACATTTAGATTTTTTTGCAATATAAGGTTTGCAGCTCTCATCAACAATTCCA
TTCTGTATTATCCAATTGAAAGCAGCCAAAGATGAACCACCGTAACACCCTAAAGCATGA
CTGCAATCAACTAGTTCTTGGACAGAAAGATTAATACTTTGATTTTTATATTTAATCATG
TAACGATCAGACAAAGTTGAGCTAGCAGCATTGGCCCAACATGAACCACAATATTTAGGT
AAATGCTGGTTTCTTAAAGGCGACAAGAAAGCTTTAGTTCTCCAATCAAACTCATCTGGA
ATGGAAGTAATATGTTGATATTCAACTTCAAGATCCTTGTCAGTTTTTGCAGCTTTGTGC
AAGAATGGTTCTCTTGTTGATTTTAAGAACTTTGATTCATTCAAATTTATAAAAGCTATG
AATACGATACATAAAAGCATTATATTAATCTTAAAATTATACAAAAAGTGTTAATTTGTT
ACTAAATATAATGGATTTGAGTTCAACAGATAAATTTTATTAGAAATTAAGGGACAGCTT
ACCAATTATTTGGAAGCAAAATATCATTTCTTATCCATATTGAAGTAAAAAAAATAATCT
TTTTTTACAACAAAAGTTGATTATATTGTTAATGTATAAATTAGTTGTATTTTGGTTAAA
ATAGTCGTGGAAAAAGTTATTTTGCTTTGCAATGGTTAGCCTTTAATACACAATTTAGAT
AAATTAATG
>TRINITY_DN31388_c0_g1_i1 len=434 path=[823:0-433] [-1, 823, -2]
AAAAAATACCGACTTAATTTAATTTATAATATCCTTATCCATTAATTACGCATGCCAATA
TATTATTATCCTAAAAAATGGTTCTTTCTGGAGGTTTTACAAATGAATTAAACACCAATA
TAGATAGAATTAATCCACATTCCAAAGATATTTTTGAATACGCGAAAAAAAATTTACAAA
GAAATGAGCATGTGGAAATGAAAATTATTGGATACTGTTCGCAAGTTGTCGCTGGAACAA
ATTATTGGATAAAACTTAATTTTGATAATGATAGTGAATGTTGGGTAAAAATGTACGAAC
CTCTACCATGTTACAATAAACCGATTGAATTTTTAGAGTTTTCTATGACATTTCCTTTCT
AAAAAAATTCAAAAATAGTTGCAAAAATATGATTTTTTTTATTTAATGTTGAAAAAAATA
GGAGGATATGAATA
>>lcl|ORF1_CathepsinD_7023
MLILVLVISFVCLSNCSKSFKVKLHKQERSLIHFLDKLNLVQKNSADPGKEDLLNFKDAQ
YYGVISLGTPVQNFTVVFDTGSSDFWVPSKRCKLSSLACKLHPKYNYKKSSTYIENGNHY
QLSYGSGTVAGILSYDDINFAGMKATKQTFGENTDMPFVPFGLAKFDGILGLGFPGLSAF
KASPVFNTILKQNPEIQPKFSFYIDRDTEDPTGGEIMFGGVDYDKLGEILVTTKVVTPLY
WTIKVDAITSDVTNICIDGCMAAVDSGTSLIAGPLHDIEILNKKLGAHKLFNNASIFKCE
DIPHLPNVDFKINNVIMTLYPNDYVIQESEVCLSAFMGIDLPSSSGISWILGDVFMGKYY

TIFDIENQTVSFGTVKNY
>>lcl|ORF1_Stefin_31388
MVLSGGFTNELNTNIDRINPHSKDIFEYAKKNLQRNEHVEMKIIGYCSQVVAGTNYWIKL
NFDNDSECWVKMYEPLPCYNKPIEFLEFSMTFPF
>>lcl|ORF2_Cathepsin_Z_55712
MLLCIVFIAFINLNESKFLKSTREPFLHKAAKTDKDLEVEYQHITSIPDEFDWRTKAFLS
PLRNQHLPKYCGSCWANAASSTLSDRYMIKYKNQSINLSVQELVDCSHALGCYGGSSLAA
FNWIIQNGIVDESCKPYIAKKSKCDKENRCTTCPNFNDPCYTVKDYKKWIIKEFGIVKIA
NMMKKEIMTNGPISCSMYASDNFLKNYKGGIYMEKANERNDHEISIVGWGLTVDEENKPL
PYWIVRNSWGQFWGEDGFFRVPTHEYSNADYSLGIGSEICDFPILF
>>lcl|ORF3_Cathepsin_L_31847
MNIFILILLSFVNCQYDKPWELHKAKYNLEFTKEENAKRFSIFFENIRKIEEHNSDPTKT
FTLKINQFGHLSNEEYKKNLANKMKVEYKPTKSNYRQAPSSDKVDWRDKNVVNPIKSQGS
CGSCYAFSTVGALESAYAIKKGKLFNLSVQQIVSCDHSNFGCDGGFPYLAIQYLKKKGIE
SWEDYPYETSLFNKCHYNHSKVLFKVKGYKNITQNEYALSHAIKTNGPVSIVIDSSPFSF
QFYDKGIYQDKYCSTEDMNHAVVLVGYSKDHKEHDYYILRNSWGTNWGIDGYMHIARFKN
NMCGVATMAVYPIV
>>lcl|ORF11_Aminopeptidase_N_22765
MGIFNKVSYISNEIQSTIETTILNDIVEIVNYVYDNDNQIVTFNLNTQLSTKNVYLLQIS
YTAKYSSTIEGLYKIFYQQNNTKKFMLATHFEPIGARSVFPCFDEPSFKATFSLFFQHPS
KTRVIANTQILKTSNLSNQISLTIFEKTVKMSTYLLAFSIGDFISQSKVTVNDISINIYA
PSYANRIEELDFAGNVTLKILPFFEKLFNQKYPYKKIDLIALPEFPSGAMENWGLLTFRN
IYIIYDDKRTAIDTKKDIISVISHELAHQWFGNLVTLDWWDNIWLNEGFANYIELIGADH
FDNRFKMLDLQVVSGWDSAIEMDSINSTHAISHQISTSTNIEELFDPISYSKGAAILRMI
HSFMGDANFTAGIRDYVETYKYKTATSENLWNILSTYTVNININGIMNNWVSKKGFPLIT
MKTVKNEENRVVITLFQHSYIALNGKDIENENTLNNFSNDLWYIPVNVIDSESPLNVRTI
LLTPISPAEIILSKNVKWFKVNAGMSGFYIVNYDEQGWKNISSVLKENHNVLTPIDRSGL
IHDAFKLACDRMISPEIVFNLILYLQNENDYLPWTMMRSKYSCLFDVIEHKSIIKLYKIL
LWRLQEHLINIDTLLKESNPLETLQQYEVALFALKYGTLPDLKDSLQKLLQRLMDKNLPG
FKYKEIRTLAILYGVDKRNATILAKLWNLYRNSTSDYDRKFLMKVIASFNEGEFSQQNIL
RVTTSENVKIQDKLSFLILIYKFCSDKMNCFTKLKESYNDLSFIMKKSVMLEFMLKVVTL
TSDKETLQQIEIFLNTLLINENSIVKNMVMEKLNEKKTGNNTTLAIKRKCQASTWISAYF
KKQLPEDLLKTCANI
>TRINITY_DN7023_c0_g2_i1 len=1266 path=[2601:0-1132 2602:1133-1168 2603:1169-1265]
[-1, 2601, 2602, 2603, -2] Match_Acc=Q76EJ2 Gene=Cathepsin_D_
AAAAATAAAGTTTTTATTTGAAGAGTCTTGTCCATTTGGGTAGTCTATAGGATGTATGTT
GTGGATATATTTTTAATATATCAACTTAAAAAAATTAAAATGCTTTAAAAGCATAGTCAA
TTAATAATTTTTGACTGTTCCAAATGAAACTGTTTGGTTTTCAATATCAAATATGGTATA
ATATTTTCCCATAAAAACATCACCTAAAATCCATGAAATACCTGATGAAGACGGTAAATC
TATTCCCATAAAAGCACTTAAACATACTTCCGATTCTTGTATAACATAATCATTTGGATA
TAATGTCATTATGACATTATTTATCTTAAAATCTACATTTGGTAAGTGAGGAATATCTTC
GCATTTAAAAATACTAGCATTGTTGAATAATTTGTGTGCTCCCAATTTTTTATTCAAAAT
TTCTATGTCATGAAGAGGTCCAGCAATTAAAGAAGTCCCACTATCAACTGCAGCCATGCA
TCCATCAATACAAATGTTCGTTACATCACTTGTAATTGCATCAACTTTAATAGTCCAATA
TAACGGGGTTACAACTTTTGTTGTAACCAAAATTTCCCCCAACTTATCATAATCAACACC
TCCAAACATTATTTCTCCTCCAGTAGGGTCTTCGGTATCTCTATCTATATAAAATGAAAA
TTTTGGTTGAATTTCAGGGTTTTGTTTCAAAATAGTGTTAAATACTGGACTAGCTTTGAA
AGCGCTTAAGCCAGGAAAACCTAAACCAAGAATACCATCAAATTTAGCTAATCCAAATGG
TACAAATGGCATATCAGTATTTTCACCAAAAGTCTGTTTAGTTGCTTTCATACCAGCAAA
ATTTATATCATCATAGGATAGTATACCTGCTACGGTTCCTGAACCATAAGATAATTGATA

ATGATTGCCGTTTTCTATATATGTAGAGGATTTTTTATAGTTATATTTGGGATGTAATTT
ACAAGCTAAACTGCTCAATTTACATCTTTTAGATGGAACCCAAAAATCAGAAGAACCTGT
GTCAAAAACAACCGTAAAATTTTGTACGGGAGTACCTAATGATATTACTCCATAATATTG
AGCATCCTTAAAATTTAGTAAATCTTCTTTTCCTGGATCAGCACTGTTTTTTTTAACATG
ATTAACCTTATCCAAAAAATGTATAAGGGATCTTTCTTGTTTATGTAGTTTAACTTTGAA
AGATTTTGAACAATTAGAAAGACATACAAAAGATATTACTAAAACCAAAATTAACATTTT
ATATAT
>TRINITY_DN22765_c0_g1_i1 len=2883 path=[6278:0-801 @6286@!:802-2646 6276:2647-2669
6277:2670-2882] [-1, 6278, 6286, 6276, 6277, -2]
TATATCACATAAAAATTTAATTTAGTTCTAACAACCCTATACTACATAATCCATATATTG
CCCACCCAATAAACTAATTTCAAATATTTGCACATGTTTTTAATAAATCTTCAGGTAATT
GTTTCTTAAAATATGCTGATATCCAAGTGGAAGCTTGACATTTTCTTTTTATTGCTAATG
TTGTATTATTTCCAGTTTTTTTTTCATTCAATTTTTCCATAACCATGTTTTTCACAATAG
AATTCTCATTTATTAACAGTGTATTTAGAAAAATTTCAATTTGTTGCAGAGTCTCTTTGT
CTGACGTTAATGTAACAACTTTTAACATAAATTCAAGCATAACACTTTTTTTCATTATAA
ATGAAAGATCATTATAAGATTCTTTTAGCTTGGTAAAGCAATTCATTTTGTCAGAGCAAA
ATTTGTAAATTAGAATTAAAAATGAAAGCTTGTCCTGGATTTTTACGTTTTCTGAAGTTG
TTACTCTTAATATATTTTGTTGTGAAAATTCTCCTTCATTAAATGAAGCTATAACTTTCA
TCAAAAACTTTCTATCATAATCGCTTGTTGAATTCCTGTACAAGTTCCACAATTTAGCCA
AGATTGTGGCGTTCCGCTTGTCAACTCCATACAAAATGGCCAATGTTCTTATTTCCTTAT
ACTTAAATCCAGGTAAATTTTTATCCATTAAACGTTGCAACAACTTTTGCAAAGAATCTT
TCAAATCTGGAAGAGTTCCGTATTTAAGAGCAAAAAGAGCTACTTCATACTGTTGTAGAG
TTTCTAATGGATTTGATTCTTTAAGAAGAGTATCTATGTTTATCAAATGTTCTTGAAGTC
TCCATAATAAAATCTTGTATAGCTTAATAATAGATTTATGCTCAATAACATCAAATAAGC
AAGAATATTTTGACCTCATCATAGTCCAAGGTAAATAATCATTTTCATTTTGTAAGTATA
AGATCAAGTTGAAGACAATTTCTGGTGATATCATTCTGTCACAAGCTAATTTGAATGCAT
CATGGATTAATCCAGATCTATCTATTGGGGTCAAAACATTATGATTTTCTTTTAAGACAC
TTGAGATGTTTTTCCATCCTTGTTCATCATAATTTACAATATAAAATCCACTCATCCCAG
CATTAACTTTGAACCATTTGACGTTTTTTGATAAGATAATTTCTGCAGGCGAAATTGGTG
TCAATAATATTGTTCTTACATTCAAGGGAGATTCACTATCGATCACATTGACTGGTATAT
ACCATAAATCGTTTGAAAAATTATTTAAAGTATTTTCATTTTCAATGTCCTTCCCATTCA
AAGCGATATAAGAGTGTTGGAATAAAGTTATCACAACTCTATTTTCTTCATTTTTTACGG
TTTTCATAGTTATCAATGGAAACCCTTTCTTGCTTACCCAATTATTCATAATCCCATTTA
TGTTTATATTCACAGTATATGTGCTTAAAATATTCCACAAGTTTTCACTTGTAGCAGTTT
TATATTTGTAAGTTTCTACATAATCTCGAATTCCTGCAGTAAAATTAGCGTCACCCATAA
AAGAATGAATCATTCTTAAAATAGCGGCTCCTTTGCTATATGAAATAGGATCAAAGAGTT
CTTCAATGTTTGTTGAAGTGCTTATTTGATGAGAAATTGCATGTGTACTATTTATACTAT
CCATTTCTATTGCTGAATCCCAACCACTAACAACCTGCAAGTCCAACATTTTAAACCTAT
TATCAAAGTGATCAGCTCCTATTAGTTCAATGTAATTAGCAAACCCTTCATTAAGCCAAA
TATTGTCCCACCAATCTAATGTAACGAGGTTTCCAAACCATTGGTGAGCCAATTCATGAG
AAATGACGGATATTATATCTTTTTTGGTGTCTATTGCAGTTCTTTTGTCATCATAAATAA
TATAAATATTTCTAAAAGTCAAAAGTCCCCAGTTTTCCATAGCACCACTTGGAAATTCTG
GTAATGCAATCAAATCAATTTTTTTGTATGGGTATTTTTGGTTAAATAGTTTTTCAAAGA
ATGGTAATATTTTTAAGGTTACGTTTCCAGCGAAATCTAGTTCCTCTATTCTGTTCGCAT
AGGATGGTGCATAAATATTTATTGAAATATCATTGACTGTGACTTTAGATTGTGAAATAA
AATCCCCAATAGAAAATGCTAAGAGATAGGTACTCATTTTGACTGTTTTTTCAAATATAG
TTAAAGAAATTTGGTTGGACAAATTACTTGTTTTTAAAATTTGAGTGTTTGCTATTACTC
TAGTTTTTGAAGGATGCTGGAAAAATAAAGAAAAAGTGGCTTTGAATGATGGCTCGTCAA
AACAGGGGAAAACAGATCTAGCGCCAATCGGCTCAAAATGAGTAGCCAACATAAATTTTT
TTGTATTATTTTGTTGGTAAAATATTTTGTACAACCCTTCTATCGTGGAAGAGTATTTTG

CTGTATATGAGATTTGTAGTAAGTATACATTTTTGGTTGACAACTGTGTATTTAGGTTAA
ACGTAACAATTTGATTATCATTATCGTAAACATAATTTACAATTTCAACGATATCATTTA
ATATAGTAGTTTCAATAGTCGATTGTATTTCATTACTGATATATGAGACTTTGTTGAAAA
TACCCATATTATTGTTGATTGTCAATGTAAATGATTTGATATTGATATGAGAGCTAATAT
GAAGGGTAATTTTAGTTACAGGTTCAAAAACGTTTAAAAATATGGTAACATTACCACTAA
AATAGTTTTGGTTATTTCCAAGGTTTAGTTGAAGGTCATAATGTGATGGTAAAATGGTTT
TATTTAATCTGTATATTGATTGAGAAATTTGATTTGAACACAAAAATAATATTAAAATTT
GAG
>TRINITY_DN55712_c3_g1_i1 len=1847 path=[5050:0-125 5051:126-149 5052:150-164 5148:165-258 5142:259-281 5134:282-326 5126:327-350 5141:351-357 5101:358-381 5133:382-722 5151:723-746 5154:747-767 5063:768-795 5150:796-902 5073:903-933 5147:934-1014 5152:1015-1035 5138:1036-1056 5131:1057-1095 5122:1096-1119 5149:1120-1180 5136:1181-1274 5130:1275-1352 5144:1353-1385 5135:1386-1441 5129:1442-1465 5153:1466-1480 5028:1481-1846] [-1, 5050, 5051, 5052, 5148, 5142, 5134, 5126, 5141, 5101, 5133, 5151, 5154, 5063, 5150, 5073, 5147, 5152, 5138, 5131, 5122, 5149, 5136, 5130, 5144, 5135, 5129, 5153, 5028, -2]
AATATTTACAAAATATCATTTAATTTATTTGCAGCAAACATTTTAATTTAATTAAAATAG
AAGATAATACAGTAGTACCTCGATCTACGCAATGGTATTCATAGCAGAATTCTGCTAGAA
CAATGAGCTGAGGTTCGAGTTACGAGATGGGAAAATTTTGAAGATAATTGATTTTGAATT
TACCGCCACAGATGGCTAGTCGGTGCGTCGGTTGCTAGTTGGAGCATCACCACCTACTGT
TGTTCACAAATAATTAAAATACAGTATCGTAACTGTGCGATTCAAGTTTACCGCCGTCTC
CTTGCCCCTCCCTTCTCCTCCAGCTCAGCCTTTGCTACCGTAGCTGATACAGTATCTAAA
CGTAAGTACCGACAGAATTTCCTGTTCAATGGCCACTCTCTTCTTTTCTCATTCTATTTA
TTATTATTTATACTGCATTAATAGTTTTGTATGAGAAAAACATGCCTTTTTATGTTTAAT
ACAGTGTTTTAGGGGAATTTTCAAGGGTAGGAACCAATTAAATTTTTTTCCCTTTATTTT
TAATGAAAATAATTTGATTGACTTACGAGATGATCGAAGAACGAGCTAGGTTATGTAACG
AATAAATCTCGTGGGTCGATTTACTACTGTATATATATATATTAAGAATTATTTGTCTAT
TAAAATCACAAAAAAATTAAAAAAACCTTATAGAATTTTAGAATAAGATTGGAAAATCAC
ATATTTCACTACCAATACCTAAAGAATAGTCTGCGTTCGAATATTCATGGGTCGGAACTC
TGAAAAATCCATCCTCACCCCAAAATTGTCCCCATGAATTTCTTACTATCCAATAAGGCA
ATGGTTTATTTTCTTCATCTACAGTCAGTCCCCAACCAACTATTGAAATTTCATGATCAT
TTCGTTCATTAGCTTTTTCCATATAAATTCCTCCTTTATAGTTTTTAAAAAGTTATCGG
ATGCATACATTGAACAAGAAATTGGACCGTTTGTCATTATCTCCTTTTTCATCATGTTAG
CAATCTTTACGATTCCAAACTCTTTAATAATCCATTTTTTATAGTCTTTTACAGTGTAAC
ATGGATCGTTAAAGTTAGGACATGTTGTGCACCTATTTTCTTTATCACATTTAGATTTTT
TTGCAATATAAGGTTTGCAGCTCTCATCAACAATTCCATTCTGTATTATCCAATTGAAAG
CAGCCAAAGATGAACCACCGTAACACCCTAAAGCATGACTGCAATCAACTAGTTCTTGGA
CAGAAAGATTAATACTTTGATTTTTATATTTAATCATGTAACGATCAGACAAAGTTGAGC
TAGCAGCATTGGCCCAACATGAACCACAATATTTAGGTAAATGCTGGTTTCTTAAAGGCG
ACAAGAAAGCTTTAGTTCTCCAATCAAACTCATCTGGAATGGAAGTAATATGTTGATATT
CAACTTCAAGATCCTTGTCAGTTTTTGCAGCTTTGTGCAAGAATGGTTCTCTTGTTGATT
TTAAGAACTTTGATTCATTCAAATTTATAAAAGCTATGAATACGATACATAAAAGCATTA
TATTAATCTTAAAATTATACAAAAAGTGTTAATTTGTTACTAAATATAATGGATTTGAGT
TCAACAGATAAATTTTATTAGAAATTAAGGGACAGCTTACCAATTATTTGGAAGCAAAAT
ATCATTTCTTATCCATATTGAAGTAAAAAAAATAATCTTTTTTTACAACAAAAGTTGATT
ATATTGTTAATGTATAAATTAGTTGTATTTTGGTTAAAATAGTCGTGGAAAAAGTTATTT
TGCTTTGCAATGGTTAGCCTTTAATACACAATTTAGATAAATTAATG
>TRINITY_DN55712_c3_g1_i2 len=1894 path=[5088:0-125 5089:126-149 5090:150-164 5127:165-267 5143:268-303 5134:304-348 5126:349-372 5141:373-379 5101:380-403 5133:404-744 5151:745-768 5154:769-789 5063:790-817 5150:818-924 5073:925-955

5147:956-1036 5066:1037-1060 5139:1061-1103 5131:1104-1142 5122:1143-1166
5149:1167-1227 5136:1228-1321 5130:1322-1399 5144:1400-1432 5135:1433-1488
5129:1489-1512 5153:1513-1527 5028:1528-1893] [-1, 5088, 5089, 5090, 5127, 5143,
5134, 5126, 5141, 5101, 5133, 5151, 5154, 5063, 5150, 5073, 5147, 5066, 5139, 5131,
5122, 5149, 5136, 5130, 5144, 5135, 5129, 5153, 5028, -2]
AATATTTACAAAATATCATTTAATTTATTTGCAGCAAACATTTTAATTTAATTAAAATAG
AAGATAATACAGTAGTACCTCGATCTACGCAATGGTATTCATAGCAGAATTCTGCTAGAA
CAATGAGCTGAGGTTCGAGTTACGAGATGGGAAAATTTTGAAGATTAATGATTTTGAATT
TACCGCCACATATGGCTAGTCGGTGCGTTGGTTGCTAGTTGGAGCATAACAATCTACTGT
TGTTTACAAATAATTAAAAGTAAACTAAAATTATAGTACAGTACAGTATCGTAACTGTGC
GATTCAAGTTTACCGCCGTCTCCTTGCCCCTCCCTTCTCCTCCAGCTCAGCCTTTGCTAC
CGTAGCTGATACAGTATCTAAACGTAAGTACCGACAGAATTTCCTGTTCAATGGCCACTC
TCTTCTTTTCTCATTCTATTTATTATTATTTATACTGCATTAATAGTTTTGTATGAGAAA
AACATGCCTTTTTATGTTTAATACAGTGTTTTAGGGGAATTTTCAAGGGTAGGAACCAAT
TAAATTTTTTTCCCTTTATTTTTAATGAAAATAATTTGATTGACTTACGAGATGATCGAA
GAACGAGCTAGGTTATGTAACGAATAAATCTCGTGGGTCGATTTACTACTGTATATATAT
ATATTAAGAATTATTTGTCTATTAAAATCACAAAAAAATTAAAAAAACCTTATAGAATTT
TAGAATAAGATTGGAAAATCACATATTTCACTACCAATACCTAAAGAATAGTCTGCGTTC
GAATATTCATGGGTCGGAACTCTGAAAAATCCATCCTCACCCCAAAATTGTCCCCATGAA
TTTCTTACTATCCAATAAGGCAATGGTTTATTTTCTTCATCTACAGTCAGTCCCCAACCA
ACTATTGAAATTTCATGATCATTTCGTTCATTAGCTTTTTCCATATAAATTCCTCCTTTA
TAGTTTTTTAAAAAGTTATCGGATGCATACATTGAACAAGAAATTGGACCGTTTGTCATT
ATCTCCTTTTTCATCATTTTAGCAATCTTTACGATTCCTAATCATTTATATTTTATATCT
ACCAAACTCTTTAATAATCCATTTTTTATAGTCTTTTACAGTGTAACATGGATCGTTAAA
GTTAGGACATGTTGTGCACCTATTTTCTTTATCACATTTAGATTTTTTTGCAATATAAGG
TTTGCAGCTCTCATCAACAATTCCATTCTGTATTATCCAATTGAAAGCAGCCAAAGATGA
ACCACCGTAACACCCTAAAGCATGACTGCAATCAACTAGTTCTTGGACAGAAAGATTAAT
ACTTTGATTTTTATATTTAATCATGTAACGATCAGACAAAGTTGAGCTAGCAGCATTGGC
CCAACATGAACCACAATATTTAGGTAAATGCTGGTTTCTTAAAGGCGACAAGAAAGCTTT
AGTTCTCCAATCAAACTCATCTGGAATGGAAGTAATATGTTGATATTCAACTTCAAGATC
CTTGTCAGTTTTTGCAGCTTTGTGCAAGAATGGTTCTCTTGTTGATTTTAAGAACTTTGA
TTCATTCAAATTTATAAAAGCTATGAATACGATACATAAAAGCATTATATTAATCTTAAA
ATTATACAAAAAGTGTTAATTTGTTACTAAATATAATGGATTTGAGTTCAACAGATAAAT
TTTATTAGAAATTAAGGGACAGCTTACCAATTATTTGGAAGCAAAATATCATTTCTTATC
CATATTGAAGTAAAAAAAATAATCTTTTTTTACAACAAAAGTTGATTATATTGTTAATGT
ATAAATTAGTTGTATTTTGGTTAAAATAGTCGTGGAAAAAGTTATTTTGCTTTGCAATGG
TTAGCCTTTAATACACAATTTAGATAAATTAATG
>TRINITY_DN55712_c3_g1_i3 len=1872 path=[5050:0-125 5051:126-149 5052:150-164
5148:165-258 5142:259-281 5134:282-326 5126:327-350 5141:351-357 5101:358-381
5133:382-722 5151:723-746 5154:747-767 5063:768-795 5150:796-902 5073:903-933
5147:934-1014 5066:1015-1038 5139:1039-1081 5131:1082-1120 5122:1121-1144
5149:1145-1205 5136:1206-1299 5130:1300-1377 5144:1378-1410 5135:1411-1466
5129:1467-1490 5153:1491-1505 5028:1506-1871] [-1, 5050, 5051, 5052, 5148, 5142,
5134, 5126, 5141, 5101, 5133, 5151, 5154, 5063, 5150, 5073, 5147, 5066, 5139, 5131,
5122, 5149, 5136, 5130, 5144, 5135, 5129, 5153, 5028, -2]
AATATTTACAAAATATCATTTAATTTATTTGCAGCAAACATTTTAATTTAATTAAAATAG
AAGATAATACAGTAGTACCTCGATCTACGCAATGGTATTCATAGCAGAATTCTGCTAGAA
CAATGAGCTGAGGTTCGAGTTACGAGATGGGAAAATTTTGAAGATAATTGATTTTGAATT
TACCGCCACAGATGGCTAGTCGGTGCGTCGGTTGCTAGTTGGAGCATCACCACCTACTGT
TGTTCACAAATAATTAAAATACAGTATCGTAACTGTGCGATTCAAGTTTACCGCCGTCTC

CTTGCCCCTCCCTTCTCCTCCAGCTCAGCCTTTGCTACCGTAGCTGATACAGTATCTAAA
CGTAAGTACCGACAGAATTTCCTGTTCAATGGCCACTCTCTTCTTTTCTCATTCTATTTA
TTATTATTTATACTGCATTAATAGTTTTGTATGAGAAAAACATGCCTTTTTATGTTTAAT
ACAGTGTTTTAGGGGAATTTTCAAGGGTAGGAACCAATTAAATTTTTTTCCCTTTATTTT
TAATGAAAATAATTTGATTGACTTACGAGATGATCGAAGAACGAGCTAGGTTATGTAACG
AATAAATCTCGTGGGTCGATTTACTACTGTATATATATATATTAAGAATTATTTGTCTAT
TAAAATCACAAAAAAATTAAAAAAACCTTATAGAATTTTAGAATAAGATTGGAAAATCAC
ATATTTCACTACCAATACCTAAAGAATAGTCTGCGTTCGAATATTCATGGGTCGGAACTC
TGAAAAATCCATCCTCACCCCAAAATTGTCCCCATGAATTTCTTACTATCCAATAAGGCA
ATGGTTTATTTTCTTCATCTACAGTCAGTCCCCAACCAACTATTGAAATTTCATGATCAT
TTCGTTCATTAGCTTTTTCCATATAAATTCCTCCTTTATAGTTTTTTAAAAAGTTATCGG
ATGCATACATTGAACAAGAAATTGGACCGTTTGTCATTATCTCCTTTTTCATCATTTTAG
CAATCTTTACGATTCCTAATCATTTATATTTTATATCTACCAAACTCTTTAATAATCCAT
TTTTTATAGTCTTTTACAGTGTAACATGGATCGTTAAAGTTAGGACATGTTGTGCACCTA
TTTTCTTTATCACATTTAGATTTTTTTGCAATATAAGGTTTGCAGCTCTCATCAACAATT
CCATTCTGTATTATCCAATTGAAAGCAGCCAAAGATGAACCACCGTAACACCCTAAAGCA
TGACTGCAATCAACTAGTTCTTGGACAGAAAGATTAATACTTTGATTTTTATATTTAATC
ATGTAACGATCAGACAAAGTTGAGCTAGCAGCATTGGCCCAACATGAACCACAATATTTA
GGTAAATGCTGGTTTCTTAAAGGCGACAAGAAAGCTTTAGTTCTCCAATCAAACTCATCT
GGAATGGAAGTAATATGTTGATATTCAACTTCAAGATCCTTGTCAGTTTTTGCAGCTTTG
TGCAAGAATGGTTCTCTTGTTGATTTTAAGAACTTTGATTCATTCAAATTTATAAAAGCT
ATGAATACGATACATAAAAGCATTATATTAATCTTAAAATTATACAAAAAGTGTTAATTT
GTTACTAAATATAATGGATTTGAGTTCAACAGATAAATTTTATTAGAAATTAAGGGACAG
CTTACCAATTATTTGGAAGCAAAATATCATTTCTTATCCATATTGAAGTAAAAAAAATAA
TCTTTTTTTACAACAAAAGTTGATTATATTGTTAATGTATAAATTAGTTGTATTTTGGTT
AAAATAGTCGTGGAAAAAGTTATTTTGCTTTGCAATGGTTAGCCTTTAATACACAATTTA
GATAAATTAATG
