# Supplementary material for: Proteases as Therapeutic Targets Against the Parasitic Cnidarian Ceratonova shasta: Characterization of Molecules Key to Parasite Virulence In Salmonid Hosts
Source: Front Cell Infect Microbiol. 2022 Jan 7;11:804864. doi: 10.3389/fcimb.2021.804864 (PMC8777295; doi:10.3389/fcimb.2021.804864)

Supplementary Figure 1.- *Ceratonova shasta* IIR cathepsin D. A) Stereo-chemical quality analysis: Ramachandran plot and statistics using PROCHECK; B) Superposition of *C. shasta* cathepsin D and tick *Ixodes ricinus* cathepsin D zymogen (PDB: 5N7N) and C) Structural alignment of *C. shasta* cathepsin D and tick cathepsin D (PDB: 5N7N) using Phyre2 modelling and UCSF Chimera for visualization (in red: aspartic catalytic sites).

A

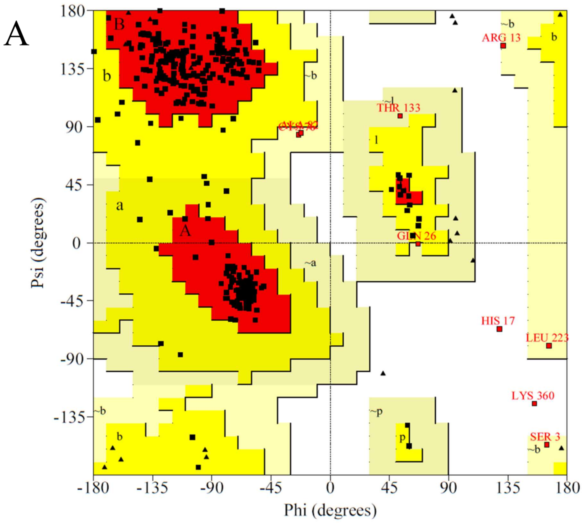

Plot statistics

Residues in most favoured regions [A,B,L]	274	87.3%
Residues in additional allowed regions [a,b,l,p]	31	9.9%
Residues in generously allowed regions [~a,~b,~l,~p]	7	2.2%
Residues in disallowed regions	2	0.6%
	----	------
Number of non-glycine and non-proline residues	314	100.0%
Number of end-residues (excl. Gly and Pro)	2	
Number of glycine residues (shown as triangles)	29	
Number of proline residues	17	

Total number of residues	362	

Based on an analysis of 118 structures of resolution of at least 2.0 Angstroms and R-factor no greater than 20%, a good quality model would be expected to have over 90% in the most favoured regions.

B

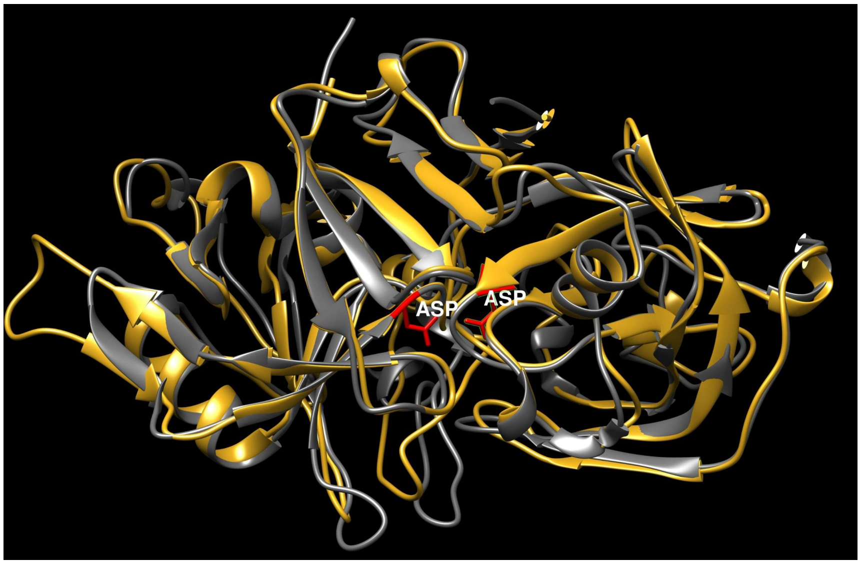

C

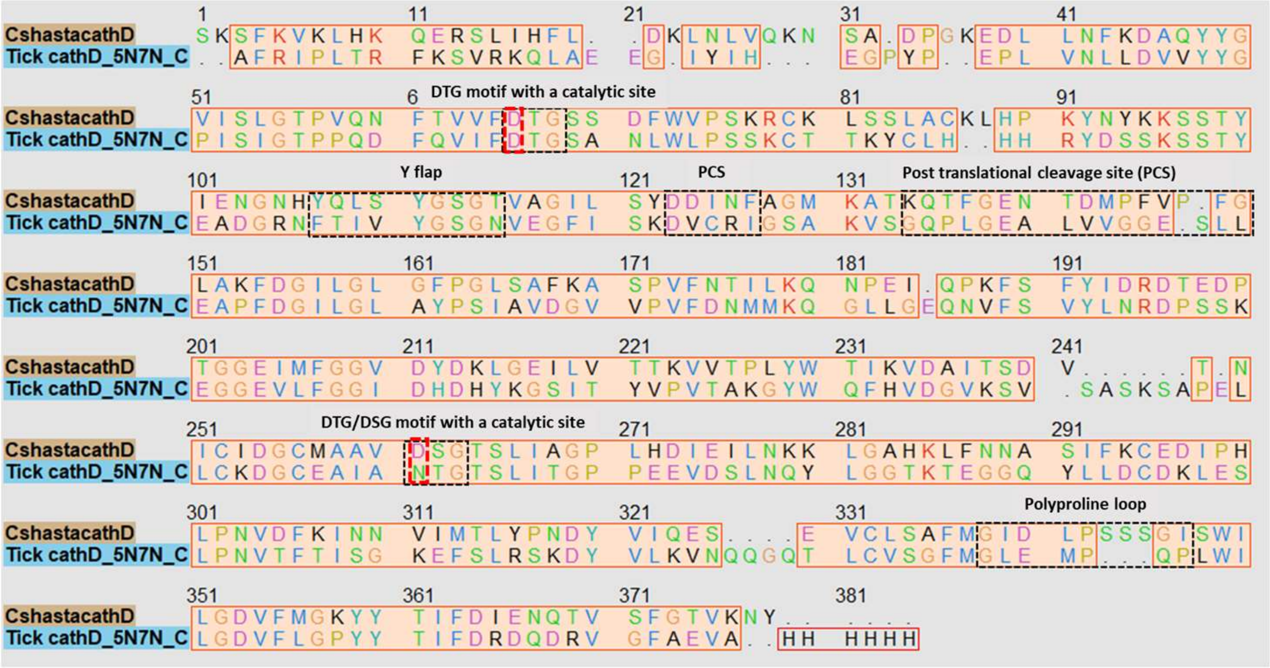

Supplementary Figure 2.- *Ceratonova shasta* IIR cathepsin L. A) Stereo-chemical quality analysis: Ramachandran plot and statistics using PROCHECK; B) Superposition of *C. shasta* cathepsin L and *Fasciola hepatica* procathepsin L (PDB: 2O6X) and C) Structural alignment of *C. shasta* cathepsin L and *F. hepatica* procathepsin L (PDB: 2O6X) using Phyre2 modelling and UCSF Chimera for visualization (in red: catalytic sites; asterisks: conserved residues in the corresponding ERFNIN and GNFD motifs).

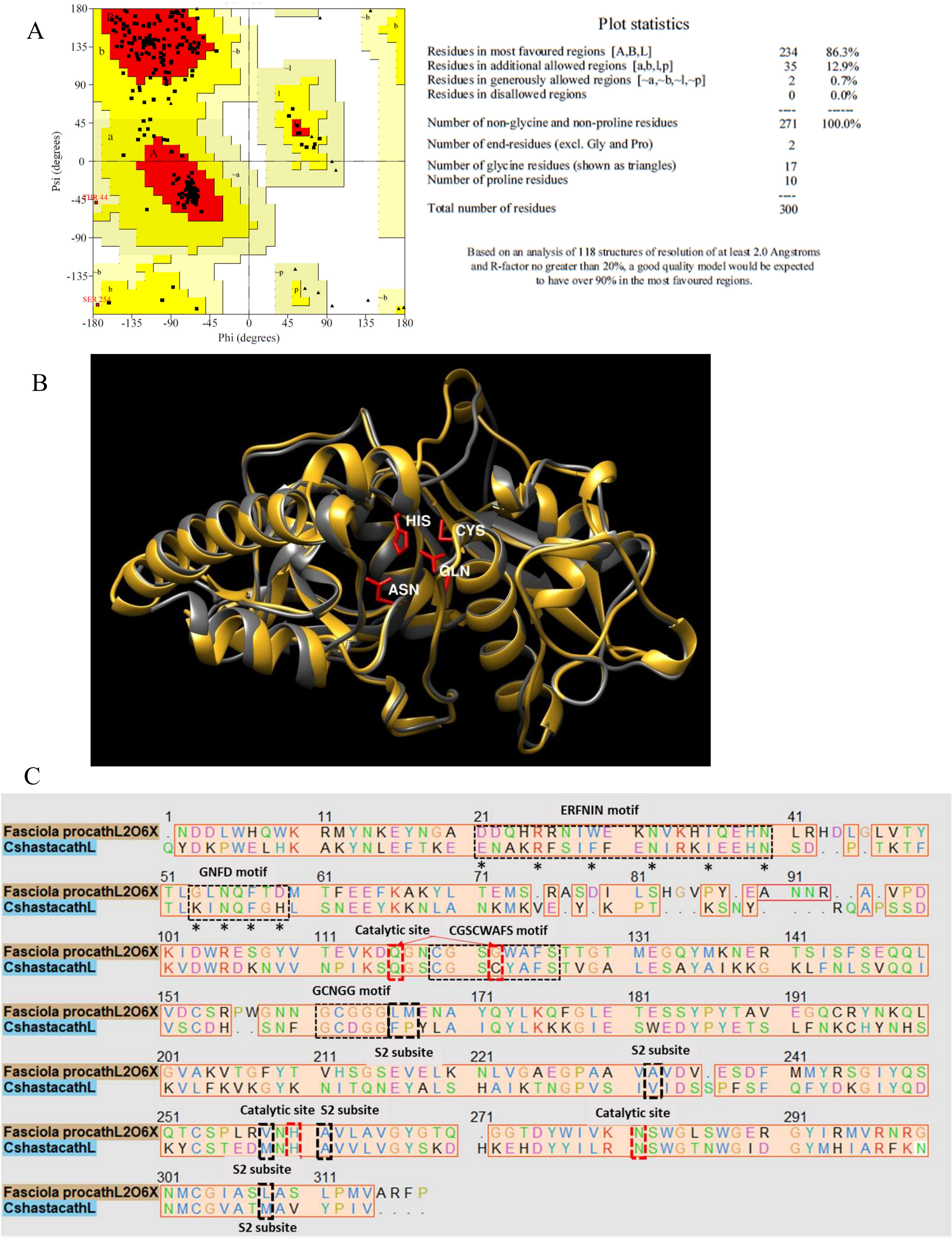

Supplementary Figure 3.- *Ceratonova shasta* IIR cathepsin Z. A) Stereo-chemical quality analysis: Ramachandran plot and statistics using PROCHECK; B) Superposition of *C. shasta* cathepsin Z and *H. sapiens* procathepsin X (PDB: 1DEU) and C) Structural alignment of *C. shasta* cathepsin Z and *H.sapiens* procathepsin X (PDB: 1DEU) using Phyre2 modelling and UCSF Chimera for visualization (in red: catalytic sites).

A

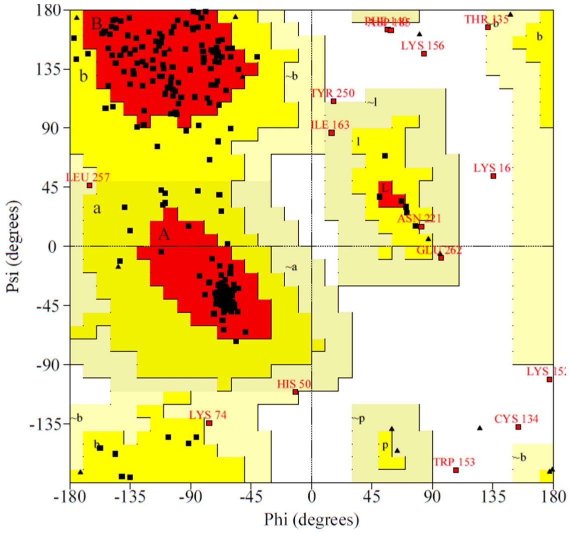

Plot statistics

Residues in most favoured regions [A,B,L]	189	78.8%
Residues in additional allowed regions [a,b,l,p]	36	15.0%
Residues in generously allowed regions [~a,~b,~l,~p]	7	2.9%
Residues in disallowed regions	8	3.3%
	----	------
Number of non-glycine and non-proline residues	240	100.0%
Number of end-residues (excl. Gly and Pro)	2	
Number of glycine residues (shown as triangles)	16	
Number of proline residues	12	

Total number of residues	270	

Based on an analysis of 118 structures of resolution of at least 2.0 Angstroms and R-factor no greater than 20%, a good quality model would be expected to have over 90% in the most favoured regions.

B

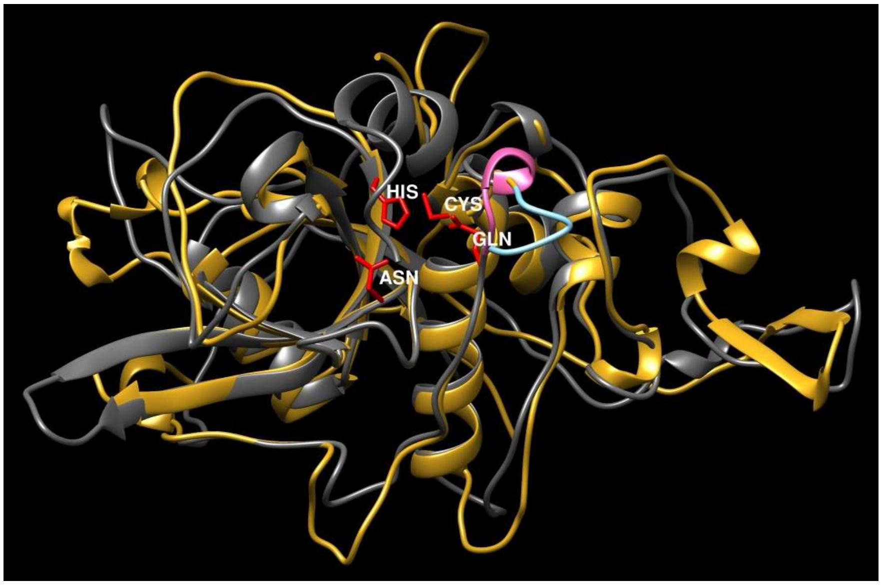

C

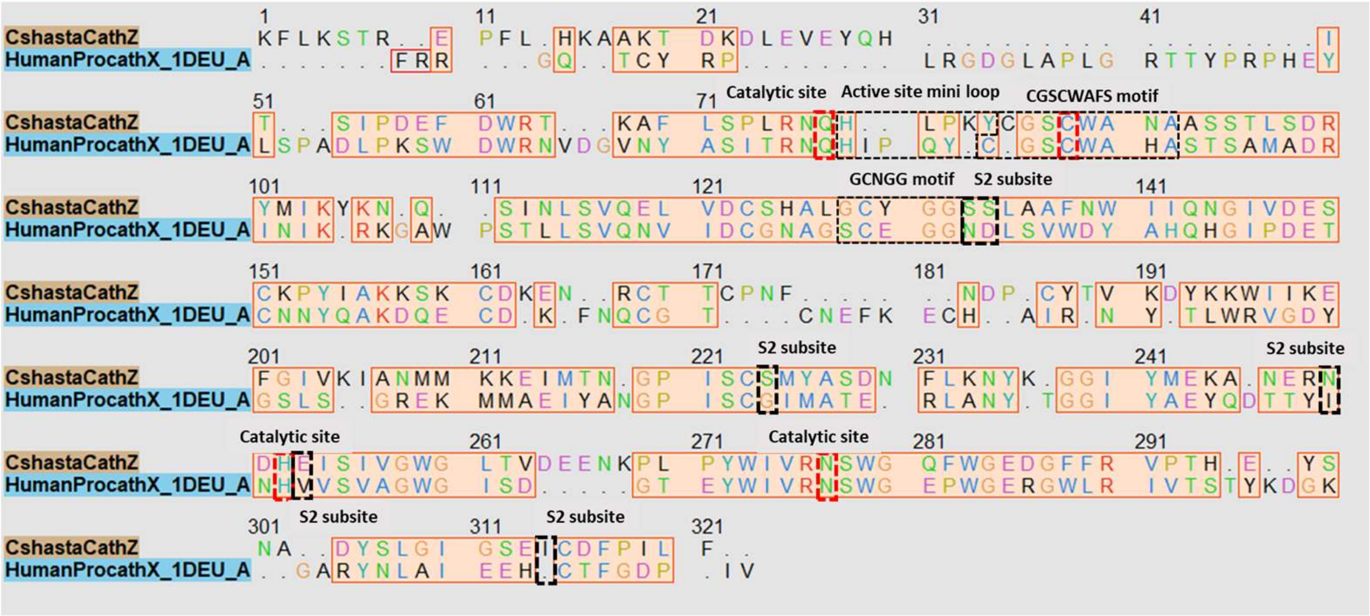

Supplementary Figure 4.- *Ceratonova shasta* IIR aminopeptidase-N. A) Stereo-chemical quality analysis: Ramachandran plot and statistics using PROCHECK; B) Superposition of *C. shasta* aminopeptidase-N and *H. sapiens* aminopeptidase-N (PDB: 4FYT) and C) Structural alignment of *C. shasta* aminopeptidase-N and *H. sapiens* aminopeptidase-N (PDB: 4FYT) using Phyre2 modelling and UCSF Chimera for visualization (in red: active sites, in yellow: Glutamyl residue required for catalytic activity, asterisks: active sites also annotated as zinc binding sites).

A

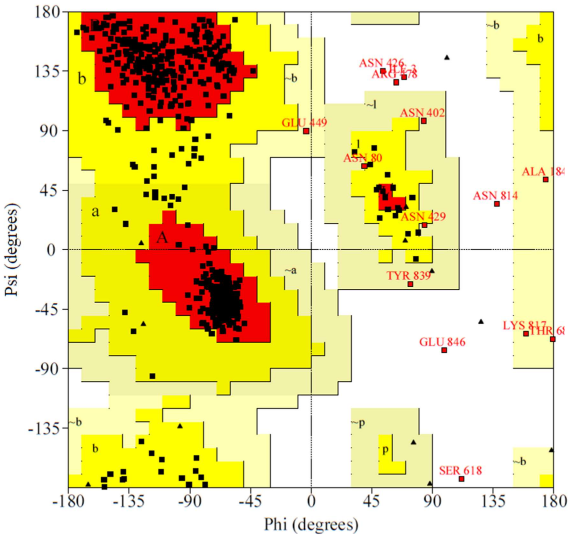

Plot statistics

Residues in most favoured regions [A,B,L]	726	90.2%
Residues in additional allowed regions [a,b,l,p]	65	8.1%
Residues in generously allowed regions [~a,~b,~l,~p]	8	1.0%
Residues in disallowed regions	6	0.7%
	----	------
Number of non-glycine and non-proline residues	805	100.0%
Number of end-residues (excl. Gly and Pro)	2	
Number of glycine residues (shown as triangles)	26	
Number of proline residues	22	

Total number of residues	855	

Based on an analysis of 118 structures of resolution of at least 2.0 Angstroms and R-factor no greater than 20%, a good quality model would be expected to have over 90% in the most favoured regions.

B

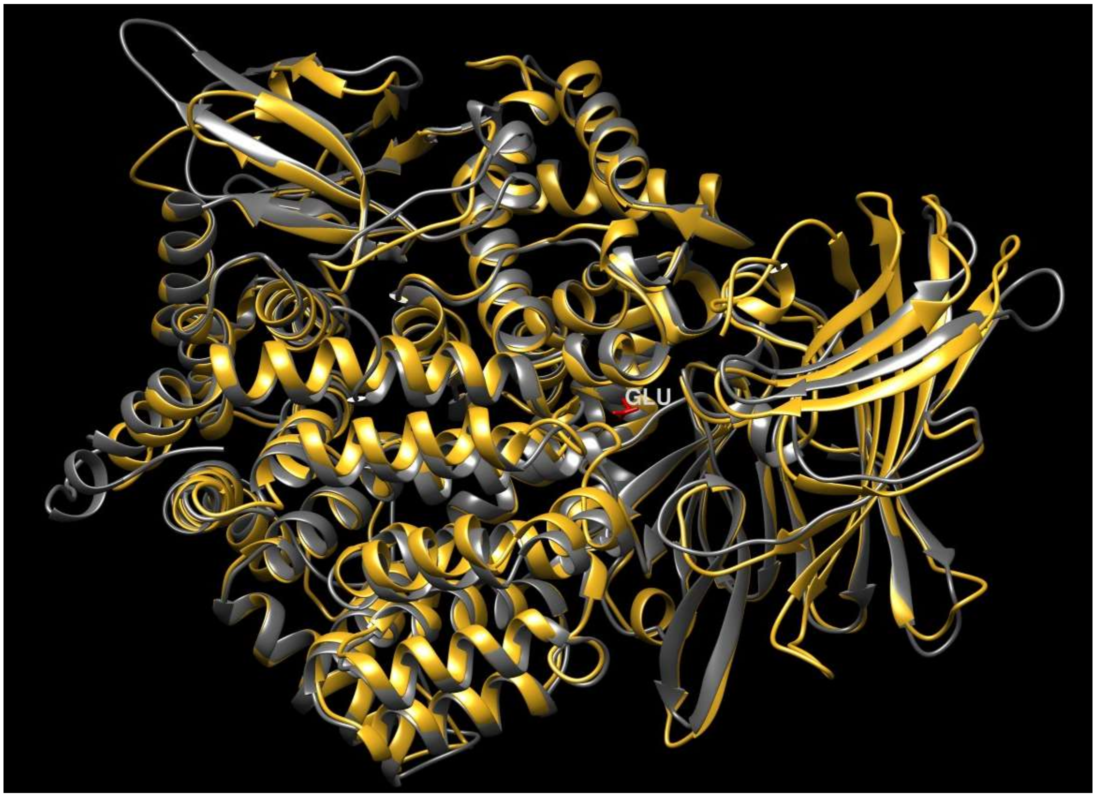

Supplementary Figure 4.- cont.

C

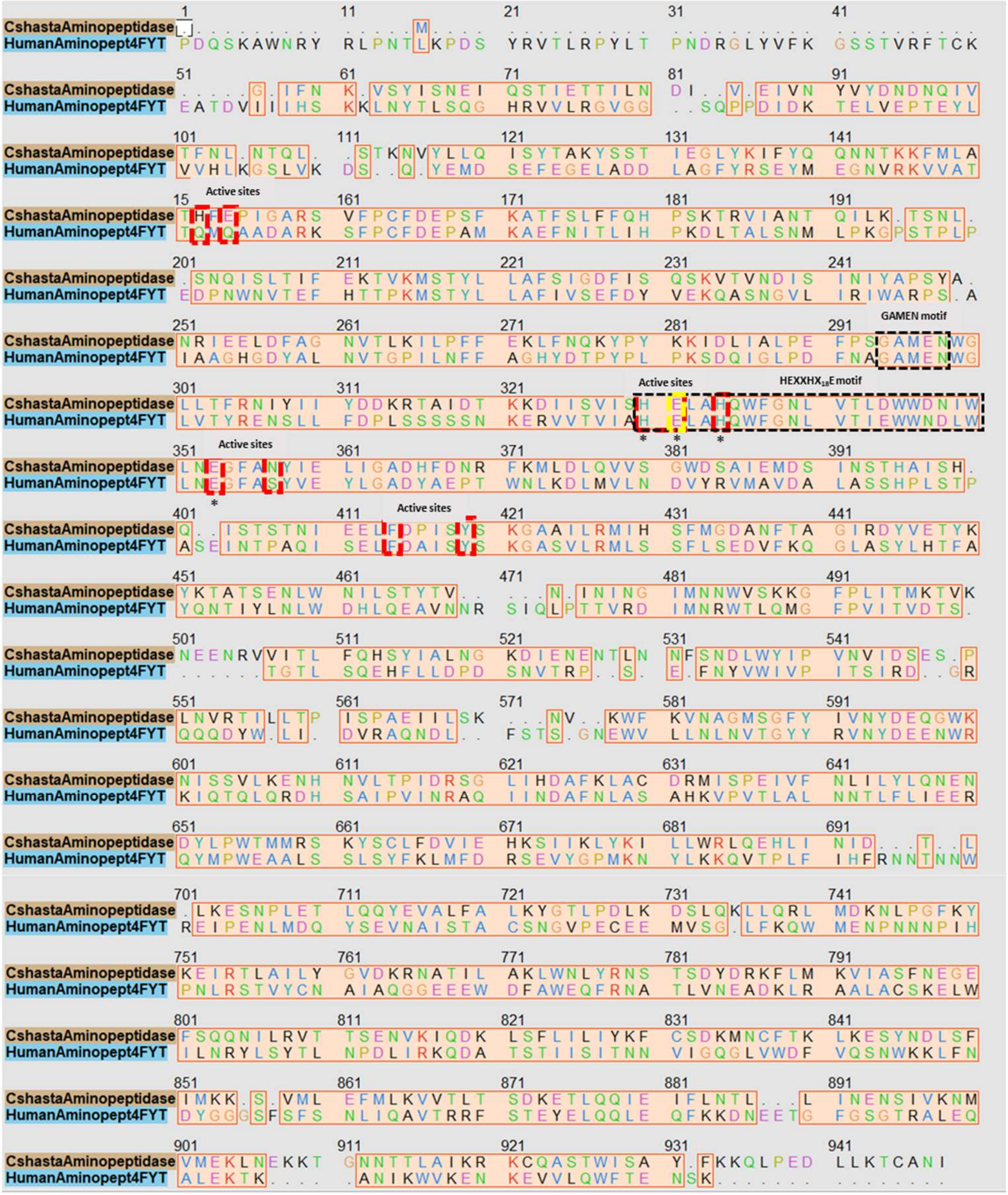

Supplementary Figure 5.- *Ceratonova shasta* IIR stefin. A) Stereo-chemical quality analysis: Ramachandran plot and statistics using PROCHECK; B) Superposition of *C. shasta* stefin and *Clonorchis sinensis* stefin (PDB: 5ZC1) and C) Structural alignment of *C. shasta* stefin and *C. sinensis* stefin (PDB: 5ZC1) using Phyre2 modelling and UCSF Chimera for visualization.

A

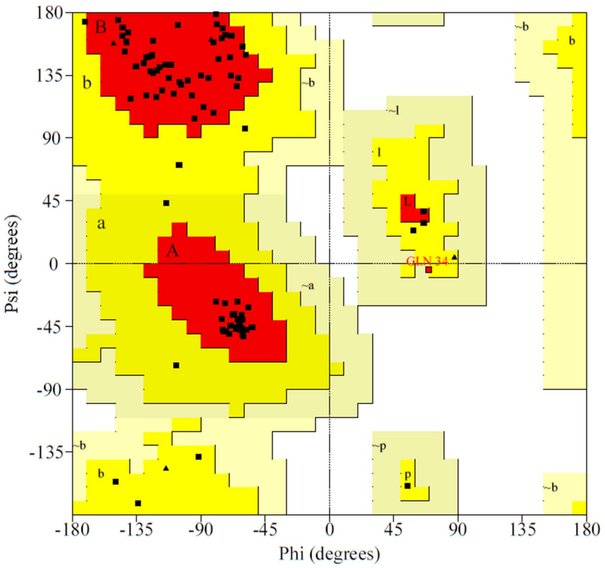

Plot statistics

Residues in most favoured regions [A,B,L]	71	85.5%
Residues in additional allowed regions [a,b,l,p]	11	13.3%
Residues in generously allowed regions [~a,~b,~l,~p]	1	1.2%
Residues in disallowed regions	0	0.0%
	----	------
Number of non-glycine and non-proline residues	83	100.0%
Number of end-residues (excl. Gly and Pro)	2	
Number of glycine residues (shown as triangles)	4	
Number of proline residues	5	

Total number of residues	94	

Based on an analysis of 118 structures of resolution of at least 2.0 Angstroms and R-factor no greater than 20%, a good quality model would be expected to have over 90% in the most favoured regions.

B

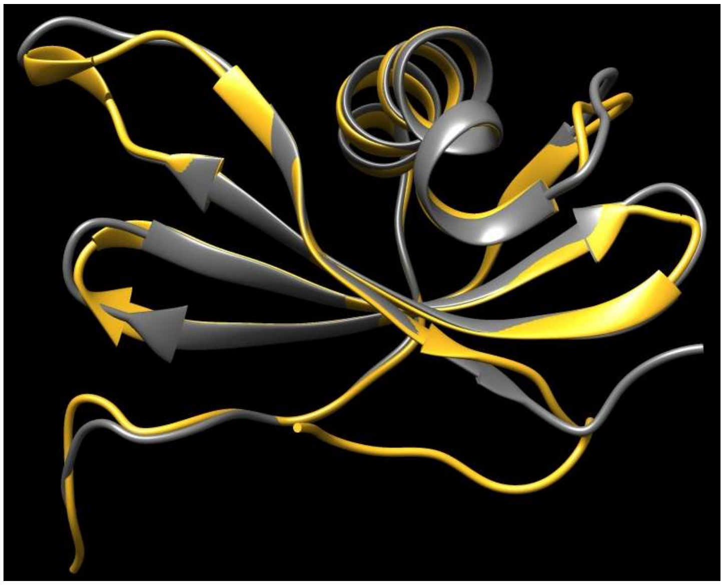

C

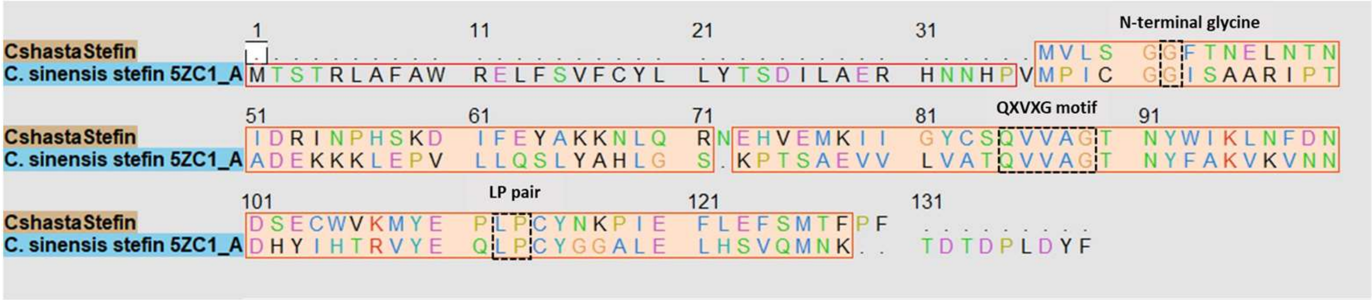

Supplement: Supplementary file 3 [file DataSheet_3.pdf]
