# Supplementary material for: Proteases as Therapeutic Targets Against the Parasitic Cnidarian Ceratonova shasta: Characterization of Molecules Key to Parasite Virulence In Salmonid Hosts
Source: Front Cell Infect Microbiol. 2022 Jan 7;11:804864. doi: 10.3389/fcimb.2021.804864 (PMC8777295; doi:10.3389/fcimb.2021.804864)
Supplement: Supplementary file 4 [file DataSheet_4.pdf]

SUPPLEMENTARY TABLES

Supplementary Table 1.- Proteases and stefin genes relative change mean ± SD using $2^{-\Delta Cq}$ method per gene, sampling day post exposure (dpe) per each genotype (0 and IIR) against their respective reference genes in the intestine (n=5 fish per genotype, otherwise indicated in the table). Statistical differences between mean fold changes at different time points (7, 15, 22, 29 dpe) were calculated using Tukey´s method for multiple comparison after one-way ANOVA or t-test for normally distributed data. For non-normally distributed data, Kruskal-Wallis with Dunn's multiple comparison was applied. Letters in parentheses indicate significant difference groupings.

Gene	Genotype	7 dpe	15 dpe	22 dpe	29 dpe	Statistical results
Cathepsin L	0	0.39 ± 0.34 (a)	2.58 ± 0.07 (b)	2.77 ± 0.59 (b)	2.81 ± 0.35 (b)	*F*=18.92; df=3; *p*<0.001
	IIR	0.30 ± 0.07 (a)	0.50 ± 0.07 (b)	0.28 ± 0.11 (ab)	0.26 ± 0.04 (ab)	*F*=10.33; df=3; *p*<0.001
Cathepsin Z	0	1.12 ± 0.41 (a)	2.92 ± 0.58 (b)	3.73 ± 0.47 (b)	2.74 ± 1.49 (ab)	*F*=9.39; df=3; *p*=0.001
	IIR	0.77 ± 0.14 (a)	1.92 ± 0.80 (b)	0.77 ± 0.10 (ab)	0.72 ± 0.19 (a)	*H*=10.68; df=3; *p*=0.014
Cathepsin D	0	0.03 ± 0.05	0.09 ± 0.03	0.09 ± 0.03	0.12 ± 0.07	*F*=2.92; df=3; *p*=0.069
	IIR	0.90 ± 0.14 (a)	1.43 ± 0.18 (b)	1.04 ± 0.22 (a)	0.76 ± 0.08 (a)	*F*=16.14; df=3; *p*<0.001
Aminopeptidase-N	0	-	-	0.004 ([1])	0.01 ± 0.01	-
	IIR	-	0.09 ([2])	0.02 ± 0.01	0.02 ± 0.01	*t*=0.23; df=8; *p*=0.827
Stefin	0	0.02 ([1])	0.01 ± 0.01	0.01 ± 0.01	0.03 ± 0.02	*F*=2.53; df=3; *p*=0.121
	IIR	0.29 ± 0.06 (a)	0.68 ± 0.37 (b)	0.23 ± 0.10 (a)	0.40 ± 0.16 (ab)	*F*=4.65; df=3; *p*=0.017

(1) Only in one genotype 0 fish this gene was quantified. (2) Only in two genotype IIR fish this gene was quantified.

Supplementary Table 2.- Proteases and stefin genes fold change mean ± SD calculated using $2^{-\Delta\Delta Cq}$ method per gene and sampling day post exposure (dpe) between *C. shasta* genotypes IIR and 0 in the intestine (n=5 fish per genotype, otherwise indicated in the table). Statistical differences between mean fold changes at different time points (7, 15, 22, 29 dpe) were calculated using Tukey´s method for multiple comparison after one-way ANOVA or t-test for normally distributed data. For non-normally distributed data, Kruskal-Wallis with Dunn's multiple comparison was applied. Letters in parentheses indicate significant difference groupings.

Gene	7 dpe	15 dpe	22 dpe	29 dpe	Statistical results
Cathepsin L	0.42 ± 0.04 (a)	0.19 ± 0.01 (b)	0.11 ± 0.02 (b)	0.18 ± 0.01 (b)	*F*=30.40; df=3; *p*<0.001
Cathepsin Z	0.77 ± 0.06 (a)	0.68 ±0.13 (ab)	0.42 ± 0.21 (ab)	0.26 ± 0.03 (b)	*H*=9.42; df=3; *p*=0.024
Cathepsin D	39.60 ± 2.79 (a)	17.96 ± 0.99 (ab)	15.83 ± 2.79 (ab)	5.25 ± 0.25 (b)	*H*=16.30; df=3; *p*<0.001
Aminopeptidase-N	-	-	4.56 ± 0.88 (*)	2.15 ± 0.53	*t*=2.34; df=8; *p*=0.0238
Stefin	18.15 ± 1.76 (*) (a)	63.61 ± 19.77 (b)	21.36 ± 4.26 (ab)	18 ± 3.32 (a)	*H*=10.43; df=3; *p*=0.015

(*)Only in one genotype 0 fish this gene was quantified.

Supplementary Table 3.**-** Genomic evidence (length, position in contig, number of introns and their position) for *C. shasta* proteases and stefin.

**Transcript (RNA sequence)**	**Gene**	**Length of RNA sequence**	**Genome contig**	**Length and position of DNA sequence**	**Number of introns**	**Positions of introns**
TRINITY_DN7023_c0_g1_i1	Cathepsin D	1,266 bp	NODE_37519_length_13598_cov_19.984262	1,310 bp (7,021-8,331)	2	750-771; 1,013-1,035 (over 1,310bp)
TRINITY_DN31847_c0_g1_i1	Cathepsin L	993bp	NODE_15179_length_31960_cov_19.503536	1,086 bp (21,842-22,928)	4	225-248; 421-445; 628-652; 927-951 (over 1,086 bp)
TRINITY_DN55712_c3_g1_i4	Cathepsin Z	1,869 bp	NODE_17068_length_2659_cov_13.214743 (not complete)	1,334 bp (1,417-2,751) partial	2	378-403; 633-660 (over 1,334 bp)
TRINITY_DN22765_c0_g1_i2	Aminopeptidase -N	2,904 bp	NODE_18414_length_42556_cov_18.465082	3,296bp (39,092-42,387)	17	504-524; 802-8291; 1,112-1,133; 1,248-1,271; 1,407-1,428; 1,495-1,518; 1,585-1,607; 1,796-1,818; 1,902-1,926; 2,058-2,081; 2,138-2,161; 2,310-2,330; 2,405-2,426; 2,532-2,552; 2,730-2,753; 2,895-2,916; 2,993-3,014 (over 3,296 bp)
TRINITY_DN31388_c0_g1_i1	Stefin	434 bp	NODE_3432_length_20735_cov_19.505232	477 bp (2,960-3,437)	2	150-169; 275-297 (over 477 bp)

Supplementary Table 4.- *C. shasta* proteases and stefin isoforms/genes found in the transcriptome assemblies, % of similarities, differences at the RNA and predicted ORF and potential relevance of these changes.

Gene name & transcript (RNA sequence)	Length of transcript	Isoforms/genes in transcriptome	Isoforms or genes assembled	% similarity at RNA level (number of different bp)	Are primers amplifying isoforms?	ORF lenght	Different aa (comparing ORF finder results)	Are these changes in regions of identified important domains?
Cathepsin D<br><br>TRINITY_DN7023_c0_g1_i1	1,266 bp	Yes, 2 genes	TRINITY_DN7023_c0_g2_i1 len=1266	99.7% (3bp)	Yes	g1 - 378 aa<br>g2 - 378 aa	Yes, 3 aa<br>g1→g2<br>L→V (position 38 aa)<br>L→H (position 40 aa)<br>Q→K (position 43 aa)	No
Cathepsin L<br><br>TRINITY_DN31847_c0_g1_i1	993 bp	-	-	-	-	314 aa	-	-
Cathepsin Z<br><br>TRINITY_DN55712_c3_g1_i4	1,869 bp	Yes, 4 isoforms	TRINITY_DN55712_c3_g1_i1 len=1847 bp<br>TRINITY_DN55712_c3_g1_i2 len=1894 bp<br>TRINITY_DN55712_c3_g1_i3 len=1869 bp	97-98.6% (57-26 bp)	Yes	i1 – 286aa<br>i2 – 179 aa<br>i3 – 179 aa<br>i4 – 286 aa	Yes, 2 aa<br>i1,i4 → i2,i3<br>IV →RY (position 176-177aa)<br>and 107 aa longer i1,i4	i1 & i4 have 107 extra aminoacids that contain a coding region with 2 active sites and 4 S2 subsites
Aminopeptidase-N<br><br>TRINITY_DN22765_c0_g1_i2	2,904 bp	Yes, 2 isoforms	TRINITY_DN22765_c0_g1_i1 len=2883	99.2%, Insert of 21 bp in i2 in 5'UTR region	Yes		No, ORF finder builds the same protease with both sequences	Probably not, splice junction in 5'UTR region
Stefin<br><br>TRINITY_DN31388_c0_g1_i1	434 bp	No	-	-	-		-	-

Supplementary Table 5.- Predicted domains, sites and conserved motifs annotated from a combination of databases (Uniprot, NCBI nr, CDD, SMART, Prosite, InterProScan) using predicted protein sequence obtained with ORFfinder (NCBI). Graphical representation can be found in Figure 2.

	**Domain site**	**Cathepsin D**	**Cathepsin L**	**Cathepsin Z**	**Aminopeptidase-N**	**Stefin**
ORF		1-378	1-314	1-286	1-855	1-94
Signal Peptide		1-16	1-14	1-16		
Domains	Inhibitor I29 domain		20-76			
	Peptidase A1 domain	61-373				
	Peptidase C1 domain		100-313	47-275		
	Peptidase M1 domain				172-398	
	ERAP-1_C domain				498-796	
	Cystatin domain					5-78
	Cystatin signature					46-61
Sites	Active site	79, 265 (Catalytic)	118, 124, 260, 281	65, 74, 222, 247	90, 92, 228-232, 264-265, 268, 287, 291, 345, 350	
	Active site miniloop			66-70		
	Active site flap (Y flap)	121-131				
	Postranslational cleavage site	136-140, 147-162				
	Polyproline loop	338-347				
	S2 subsites		166-167, 232, 258, 261, 308	116-117, 196, 220, 223, 279		
	Zinc binding sites				264, 268, 287	
Motifs, residues	Catalytic motif	79-82, 265-268				
	ERFNIN motif		35-54			
	GNFD motif		64-70			
	CGSCWAFS motif		121-128	71-78		
	GCNGG motif		161-165	111-115		
	GAMEN motif				228-232	
	$HEXXHX_{18}E$ motif				264-284	
	N-terminal glycine residue					6
	QXVXG motif					49-53
	LP par					76-77

Supplementary Table 6.- List of templates used to model the final 3D structures of *C. shasta* target proteins using Phyre2. The template structures used for superposition with *C. shasta* proteins are grey-shaded.

***C. shasta* protein**	**Template**	**PDB database**	**Alignment coverage**	**Identity**	**Confidence level**
Cathepsin D (TRINITY_DN7023_c0_g1_i1)	Tick *Ixodes ricinus* cathepsin D zymogen	5N7N	98%	36%	100.0%
	Mouse renin	1SMR	91%	36%	100.0%
	Porcine pepsinogen	3PSG	98%	38%	100.0%
	Human pepsin	1PSO	89%	40%	100.0%
	Rat prorenin	5MLG	98%	36%	100.0%
	Barley prophytepsin	1QDM	98%	40%	100.0%
Cathepsin L (TRINITY_DN31847_c0_g1_i1)	*Ambrosia artemisiifolia* Amb a 11 cysteine protease	5EGW	99%	35%	100.0%
	Ambrosia artemisiifolia Amb a 11 cysteine protease	5EF4	99%	35%	100.0%
	Human procathepsin K	7PCK	99%	39%	100.0%
	Human procathepsin S	2C0Y	99%	35%	100.0%
	Procathepsin L1 from *Fasciola hepatica*	2O6X	99%	35%	100.0%
	Procathepsin L3 of *Tenebrio molitor*	3QT4	99%	37%	100.0%
Cathepsin Z (TRINITY_DN55712_c3_g1_i4)	*Ambrosia artemisiifolia* Amb a 11 cysteine protease	5EGW	99%	32%	100.0%
	Human procathepsin X	1DEU	99%	40%	100.0%
	Bromelain precursor	6U7D	99%	30%	100.0%
	Ambrosia artemisiifolia Amb a 11 cysteine protease	5EF4	99%	30%	100.0%
	Human procathepsin K	7PCK	99%	30%	100.0%
	Procathepsin L1 from *Fasciola hepatica*	2O6X	99%	31%	100.0%
Aminopeptidase -N (TRINITY_DN22765_c0_g1_i2)	Human endoplasmic reticulum aminopeptidase 2	3SE6	96%	29%	100.0%
	Alanyl aminopeptidase N of *Anopheles gambiae*	4WZ9	94%	25%	100.0%
	Human endoplasmic reticulum aminopeptidase 1	2XDT	97%	29%	100.0%
	Human aminopeptidase A	4KXD	96%	27%	100.0%
	Human aminopeptidase N	4FYT	97%	28%	100.0%
	Porcine aminopeptidase N	4F5C	97%	27%	100.0%
Stefin (TRINITY_DN31388_c0_g1_i1)	*Clonorchis sinensis* stefin 1	5ZC1	94%	27%	100.0%
	Human stefin A	1NB5	92%	22%	100.0%
